# Not Always Sticky: Specificity of Protein Stabilization by Sugars Is Conferred by Protein–Water Hydrogen Bonds

**DOI:** 10.1021/jacs.3c08702

**Published:** 2023-10-16

**Authors:** Gil I. Olgenblum, Neta Carmon, Daniel Harries

**Affiliations:** The Fritz Haber Research Center, and the Harvey M. Kruger Center for Nanoscience & Nanotechnology, Institute of Chemistry, The Hebrew University, Jerusalem 9190401, Israel

## Abstract

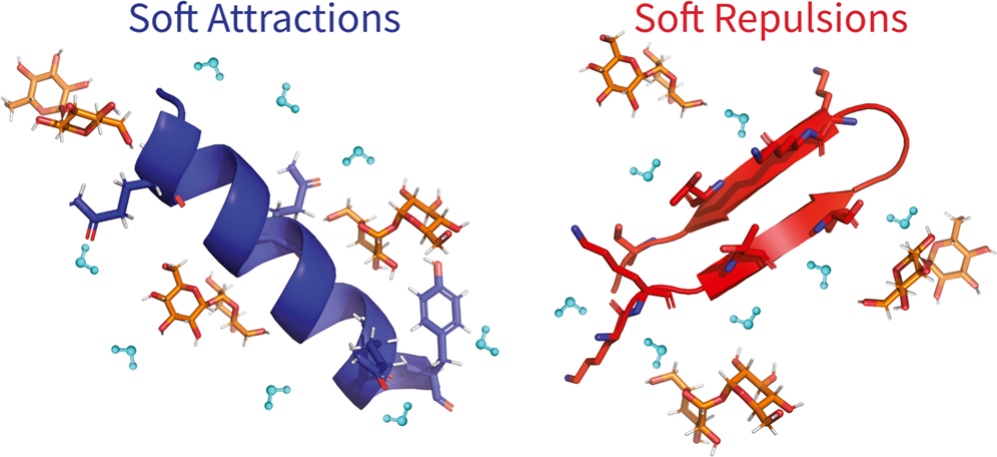

Solutes added to buffered solutions directly impact protein folding. Protein stabilization by cosolutes or crowders has been shown to be largely driven by protein–cosolute volume exclusion complemented by chemical and soft interactions. By contrast to previous studies that indicate the invariably destabilizing role of soft protein–sugar attractions, we show here that soft interactions with sugar cosolutes are protein-specific and can be stabilizing or destabilizing. We experimentally follow the folding of two model miniproteins that are only marginally stable but in the presence of sugars and polyols fold into representative and distinct secondary structures: β-hairpin or α-helix. Our mean-field model reveals that while protein–sugar excluded volume interactions have a similar stabilizing effect on both proteins, the soft interactions add a destabilizing contribution to one miniprotein but further stabilize the other. Using molecular dynamics simulations, we link the soft protein–cosolute interactions to the weakening of direct protein–water hydrogen bonding due to the presence of sugars. Although these weakened hydrogen bonds destabilize both the native and denatured states of the two proteins, the resulting contribution to the folding free energy can be positive or negative depending on the amino acid sequence. This study indicates that the significant variation between proteins in their soft interactions with sugar determines the specific response of different proteins, even to the same sugar.

## Introduction

The thermodynamic stability of proteins in the native state is greatly influenced by their solvating environment. Changes in external parameters including temperature,^1−4^ pressure,^5−9^ and even chemical activities like pH^10−13^ can direct proteins toward or away from their native state. It is therefore not surprising that the concentration and chemical identity of added solutes, sometimes termed cosolutes, also modulate the native state stability. Indeed, in a magnificent feat of adaptation, many biological life forms modulate their protein stability in response to external duress, including desiccation, salinity, and changes in temperature, by the synthesis and accumulation of large amounts of sugars and polyols. Examples include trehalose in tartigrades and yeast,^14−16^ sucrose in plant seeds,^17^ and glycerol in fungi and insects.^18^

An important consequence of protein–cosolute interactions is the emergence of solvent-mediated forces that are facilitated by cosolute inclusion or exclusion from the protein’s interface.^19−27^ Cosolute exclusion or “crowding” drives a depletion interaction that favors the protein’s more compact native state; conversely, cosolute inclusion induces protein destabilization.^28−30^ Increased native state stability in the presence of cosolutes corresponds to a negative change in the folding free energy, ΔΔ*G*^0^(*c*) = Δ*G*^0^(*c*) – Δ*G*^0^(0), where Δ*G*^0^(0) is the folding free energy in pure or buffered solvent (water) and Δ*G*^0^(*c*) is its value at a cosolute concentration *c*.

The degree of cosolute exclusion or inclusion depends on the identity of both protein and cosolute since these determine the interplay of steric and soft (i.e., weak, nonspecific) chemical interactions among the protein, cosolute, and solvent molecules. A measure of cosolute exclusion from a protein surface is the preferential interaction parameter, Γ_C_, or the related preferential solvation, Γ_S_, which correspond to the average excess or deficit number of cosolute (C) or solvent (S) molecules near the protein compared to their value in the bulk.^26,31−34^ The stabilization efficacy of a cosolute can be determined by the difference between the preferential solvation of the native (N) and denatured (D) states, ΔΓ_S_ = Γ_S,N_ – Γ_S,D_. This ΔΓ_S_ is proportional to the commonly reported m-value,^26,35−37^ typically defined as the slope of the folding free energy with concentration. Thus, a more negative value of ΔΓ_S_ corresponds to a larger stabilizing effect of the cosolute on the native state.

Several molecular models have been suggested to quantify the stabilizing contribution of cosolutes in terms of protein–cosolute steric or excluded volume interactions. Of these, most notable are the Asakura and Oosawa model^38,39^ (AOM) and scaled particle theory (SPT).^40−45^ The AOM is especially illuminating since it directly links ΔΔ*G*^0^ to changes in cosolute–protein excluded volume upon folding, Δ*V*_ex_, and osmotic pressure, Π, using a simple relation: ΔΔ*G*_AOM_^0^ = *Π*Δ*V*_ex_. Since for low cosolute concentration in the van’t Hoff regime Π ∼ *c*, the AOM predicts a linear relation between the folding free energy and cosolute concentration, as indeed often observed in folding (or unfolding) experiments.^46−51s^ Importantly, deviations from this linear dependence in cosolute concentration have also been observed.^52,53^ Although these deviations can be accounted for to some extent by nonideal contributions to the osmotic pressure or by employing the formalism of SPT,^43,44,54^ designing a model that consistently fits the folding free energy in the presence of different cosolutes and over a wide concentration range has remained challenging.

Beyond steric interactions, it is now widely appreciated that soft interactions also contribute significantly to ΔΔ*G*^0^. The current standard view of the soft interaction contribution is that it destabilizes proteins and opposes the stabilizing excluded volume term.^44,55−60^ Interestingly, several studies have indicated that soft interactions can even be stabilizing.^2,61^ Subsequently, recent theoretical strategies usually incorporate this more elusive protein–cosolute soft interaction by introducing a weak binding term to the protein free energy,^56,57^ and the latest models also consider repulsive soft interactions.^62−64^

The challenge of designing a theoretical model for protein crowding further increases when trying to fit not only the free energy but also its temperature dependence. This temperature dependence is of special interest because it contains additional information about the entropic and enthalpic contributions of soft interactions to the protein folding mechanism. Specifically, the enthalpic, Δ*H*^0^, and entropic, Δ*S*^0^, contributions to folding act as a thermodynamic fingerprint for the protein–cosolute and cosolute–solvent interactions that should be accounted for by any proposed model. Indeed, the mechanism of protein stabilization by cosolutes is now known to vary considerably between different cosolutes, ranging from an entropically dominated mechanism that is governed by excluded volume interactions to an enthalpically dominated mechanism governed by soft interactions, where entropic contributions are usually unfavorable.^2,50,53,54,65−68^

To quantitatively unravel the thermodynamic mechanism of cosolutes’ stabilization of proteins, and to shed light on the origin of the enthalpic and entropic contributions to the folding free energy, we have previously developed a mean-field model for molecular crowding.^53,64^ The model is based on the Flory–Huggins (FH) theory for binary solutions,^69,70^ in which the cosolute and solvent mixing free energy, Δ*G*, is determined by the cosolute size, concentration, and its nonideal interactions with the solvent. Cosolute size is treated by the excluded volume parameter, ν, representing the ratio between the cosolute and solvent partial molar volumes. The cosolute–solvent interactions are represented by the well-known FH parameter, χ, which describes the free energy associated with nonexcluded volume contributions to nonideal mixing between cosolute and solvent.^69,71^ The cosolute–solvent mixing free energy is then given by
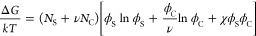
1where *N*_S_ and *N*_C_ are the number of solvent (water) and cosolute molecules, ϕ_S_ and ϕ_C_ are the solvent and cosolute volume fractions, *k* is the Boltzmann constant, and *T* is temperature.

In our crowding model, the ternary protein–cosolute–solvent mixture is divided into protein and bulk domains that are governed by the FH’s excluded volume and nonideal cosolute–solvent mixing interactions, together with additional soft protein–cosolute interactions. Each interaction term involves one of three model parameters: ν, χ, or ε. The additional parameter, ε, corresponds to the gain or loss of free energy associated with replacing protein–water contacts with protein–cosolute contacts.

Importantly, the model parameters are determined independently of each other, and ν and χ are resolved from experiments of solvent–cosolute binary solutions that do not involve protein, rendering the likelihood of model overfitting low. Moreover, the enthalpic and entropic contributions to the nonideal solvent–cosolute mixing interactions are determined by fitting the temperature dependence of binary solution osmotic pressure, and thus are also resolved independently from the contributions of the soft protein–cosolute interactions. By independently resolving the enthalpic and entropic contributions of the important nonideal mixing and soft interactions, our model is able to account for the thermodynamic origin of the measured enthalpy and entropy of folding due to the presence of cosolutes. For example, we have recently used this methodology to show that the experimentally observed enthalpic stabilization of SH3 domain protein by large polyethylene glycol (molecular weight over 1000 g per mole) is mostly due to the heat generated when solvent and cosolute at the protein interface are released and mixed with the bulk upon folding, and are not due to soft protein–cosolute interactions.^53^ This contribution of solvent release and nonideal mixing is typically neglected in other models. Full model details are in Section S1.2 in the SI, and a publicly available implementation is available from Github.^72^

**Figure 1:**
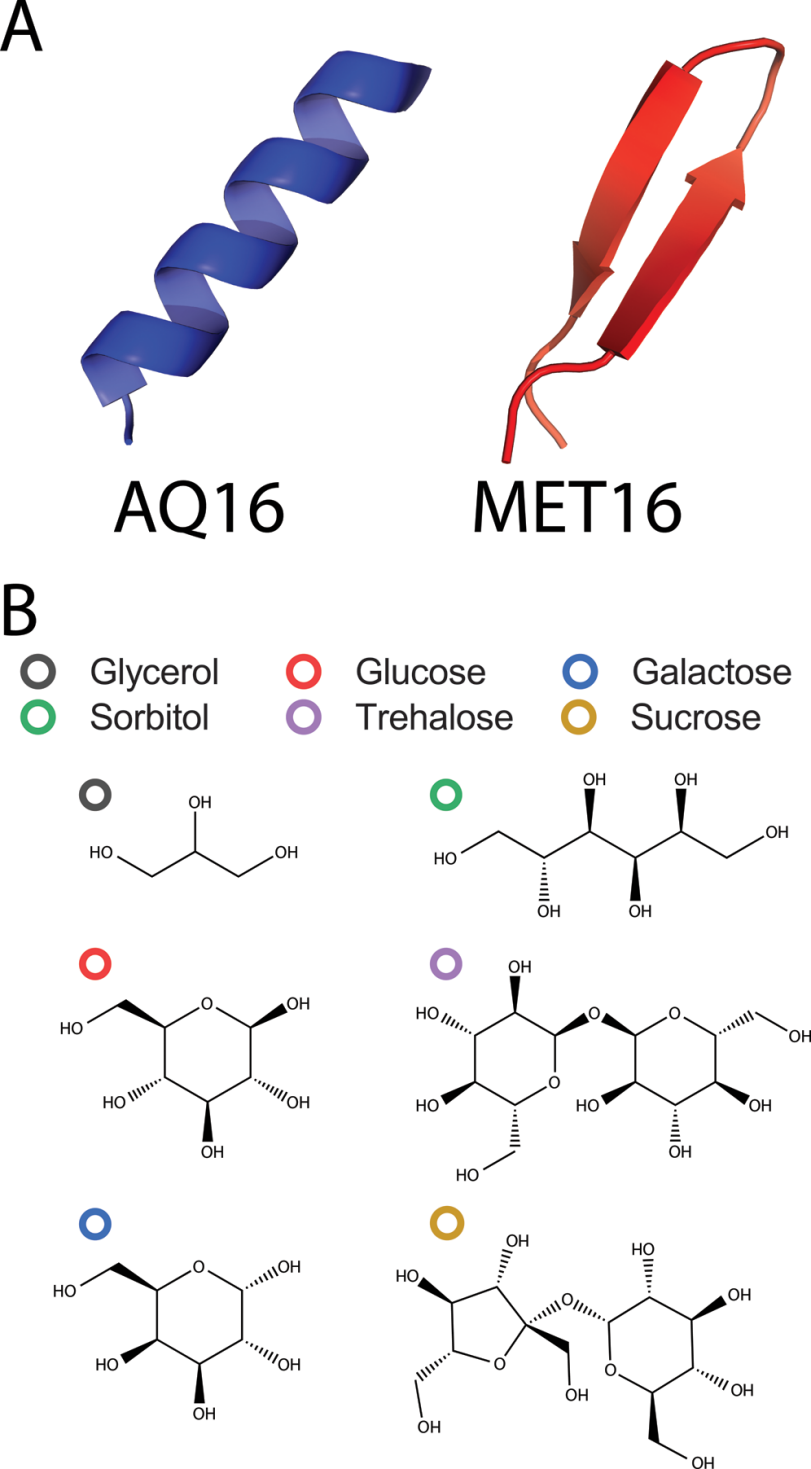
Schematic of model proteins and cosolutes. (A) Schematic of the miniproteins’ native secondary structures. (B) Chemical structures of carbohydrates.

Here, we study the effect of mono- and disaccharides, as well as polyols on protein stability (see Figure 1B for cosolute chemical structures). Using circular dichroism (CD) spectroscopy, we follow the folding thermodynamics of two distinct model miniproteins, each composed of 16 amino acid residues. The two miniproteins, named AQ16 and MET16, differ in their native secondary structures, Figure 1A (sequences are in Section S1.1 in the SI). MET16 folds into a β-hairpin,^73,74^ whereas AQ16 folds into an α-helix.^75^ In aqueous solutions at equilibrium, both AQ16 and MET16 populate two distinct states: a compact native state, *N*, and a more extended denatured state, *D*. Both miniproteins have marginal native state stability at pH 7. This relative instability allows, in turn, the measurement of even small shifts in equilibrium as a response to cosolute concentration and temperature changes.

By fitting our model to the experimentally determined folding free energies, we find that the effect of the protein–cosolute soft interactions is very different for the two miniproteins, even for the same carbohydrate. Specifically, soft interactions are stabilizing toward MET16 in the presence of most sugars, but for all sugars, they are consistently destabilizing toward AQ16. Molecular dynamics simulations suggest that the stabilization of MET16 and destabilization of AQ16 arise from disproportionately weakened hydrogen bonds of the native and denatured states with neighboring water molecules in the presence of sugars.

## Results and Discussion

### Sugars and Polyols Increase Protein Stability

**Figure 2:**
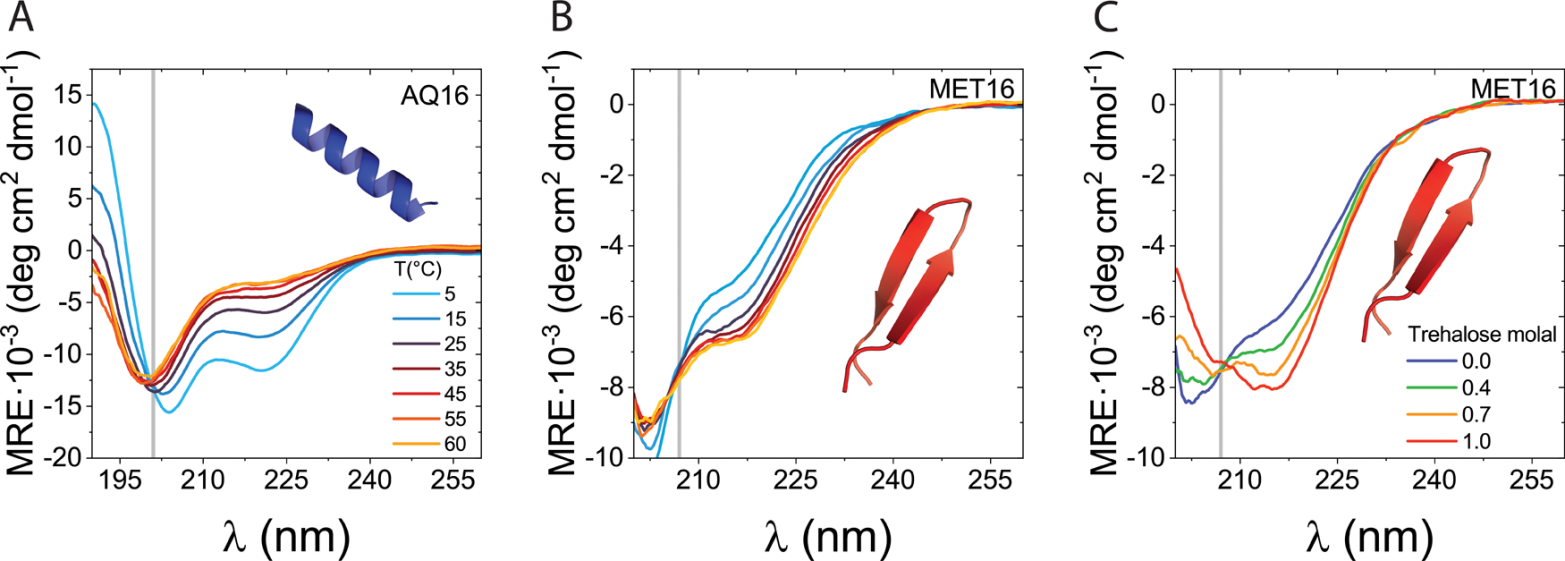
CD spectra of model miniproteins at different temperatures and cosolute concentrations. (A) CD spectra of AQ16 at different temperatures at pH 7. (B) CD spectra of MET16 at different temperatures at pH 7. (C) CD spectra of MET16 at different trehalose molalities at *T* = 298 K. Isodichroic points are denoted by vertical gray lines. Ellipticity is reported in units of mean residue ellipticity (MRE).

We followed the native state stability of AQ16 and MET16 in the presence and absence of different carbohydrates using CD spectroscopy. Figure 2A,B shows CD spectra for the miniproteins at different temperatures in buffered solution and in the absence of sugar. The isodichroic points for AQ16 (at 201 ± 1 nm) and MET16 (at 207 ± 1 nm) strongly support two-state *D* ⇌ *N* equilibrium (see gray lines in Figure 2A, B). The CD spectra for AQ16, Figure 2A, show two minima (at 208 and 222 nm) associated with the folded α-helix structure, whereas the spectra for MET16, Figure 2B, show a single minimum at 215 nm, typical of β-sheets.

The native state stability of the two miniproteins is affected differently by temperature. While the depth of the two minima of AQ16’s native state diminishes with temperature, the minimum for MET16 becomes deeper (Figure 2A,B). These trends with temperature correspond to AQ16 destabilization and MET16 stabilization with increasing temperature. An increase in native state stability with temperature, as observed for MET16, is frequently associated with the dominance of entropic hydrophobic forces that favor the more compact folded state.^76−79^ By contrast, AQ16’s native state destabilization with temperature indicates heat release upon folding. These folding mechanisms are not mutually exclusive, and many proteins may change dominance from one mechanism to the other depending on temperature^4,25,80^ or solvating environment.^81^ The difference in the folding mechanism between the two miniproteins reflects the different interactions of each miniprotein with its solvating environment and the different internal protein interactions.

Next, we followed the impact of the added sugars on protein stability. Figure 2C shows, as an example, MET16’s CD spectra for different concentrations of the disaccharide trehalose at 25 °C (the corresponding results for AQ16 are in Section S5 in the SI). The deepening of the minimum at 215 nm with increasing sugar concentration indicates further stabilization of the native state. Importantly, MET16’s and AQ16’s isodichroic points are unchanged with temperature and cosolute variations within measurement error (gray line, Figure 2C for MET16 and Figure S5 for AQ16), suggesting that the relative fraction but not the structures of native and denatured populations change in the presence of carbohydrates for both miniproteins.

**Figure 3:**
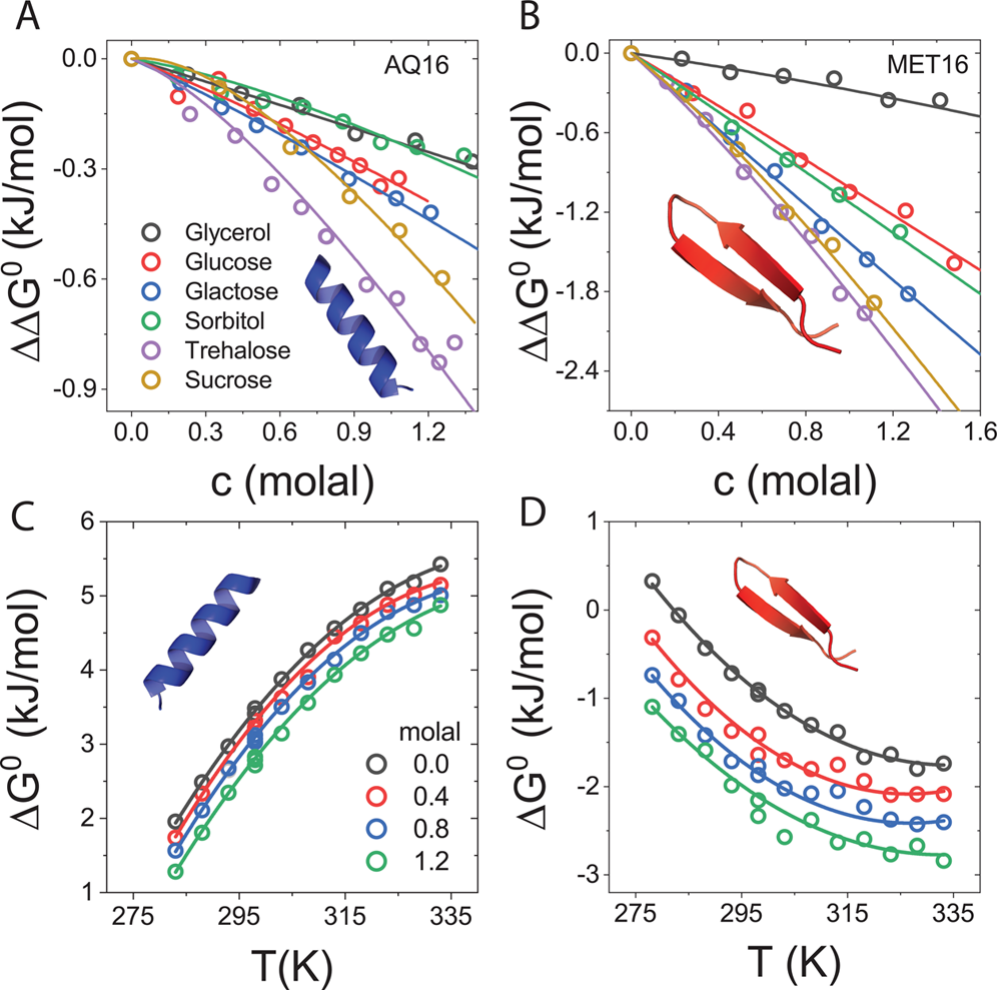
Effect of sugars and polyols on protein folding free energy. (A) AQ16’s change in folding free energy versus cosolute concentration. (B) MET16’s change in folding free energy versus cosolute concentration. For both miniproteins, data are for *T* = 298*K* and curves are fits to the model; fit details are in Section S1.5 in the SI. (C) AQ16’s folding free energy versus temperature for different trehalose concentrations. (D) MET16’s folding free energy versus temperature for different trehalose concentrations. Curves are fits to the integrated form of the van’t Hoff equation, eq 3.

The relative fractions of native and denatured states are determined from the CD measurements and used to resolve the change in folding free energy due to cosolutes, ΔΔ*G*^0^, versus cosolute concentration; see details in Section S1.5 in the SI. All cosolutes stabilize the miniproteins native state, Figure 3A,B. However, the stabilization efficacy of the cosolutes does not simply correlate with their molecular size, as prescribed by the AOM and other theories based on excluded volume considerations alone. Perhaps the most prominent is the stabilizing effect of the monosaccharide galactose on MET16, which rivals that of the much larger disaccharide sucrose. In contrast, the stabilizing effect of glycerol and the much larger sorbitol on AQ16’s native state are comparable.

Figure 3C,D shows protein folding free energies versus temperature at different trehalose concentrations. The different folding mechanisms of the miniproteins (entropically versus enthalpically driven) manifest in opposite slopes of Δ*G*^0^ with temperature since AQ16 is destabilized and MET16 is stabilized with increasing temperature. For both proteins, the addition of trehalose shifts the curves to more negative values, indicating protein stabilization. This trend is seen also for other sugar.

### Excluded Volume Cannot Account for Specificity of Protein Stabilization by Sugar

**Figure 4:**
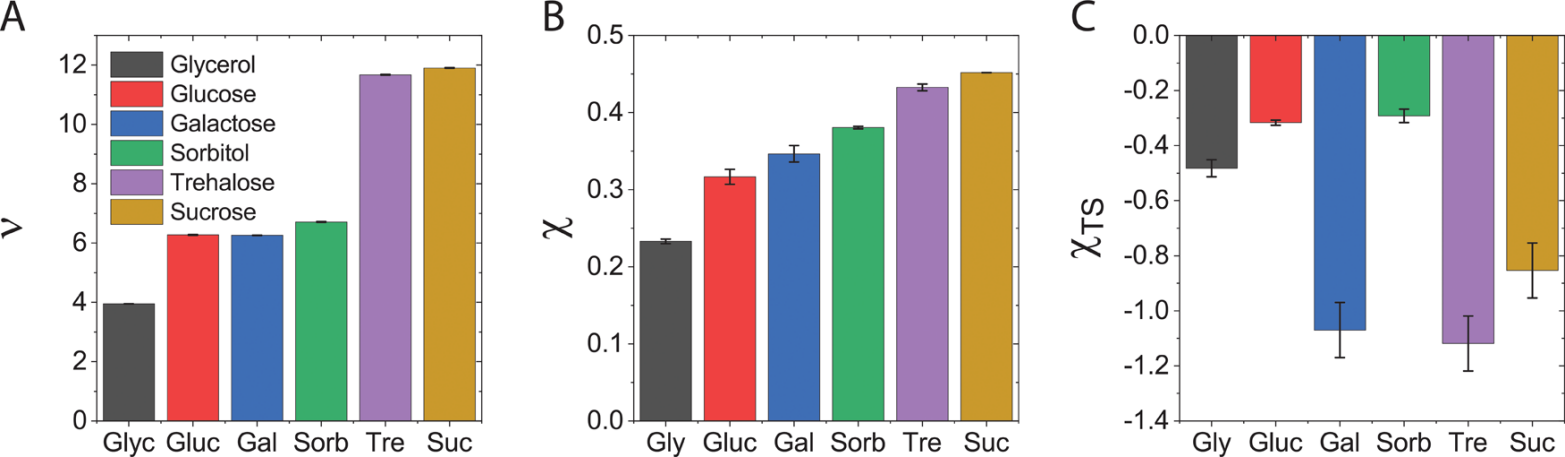
Cosolute excluded volume parameter, ν, and nonideal cosolute–solvent interaction parameter, χ, extracted from binary solution density and water activity measurements. (A) Values of ν, (B) values of χ, and (C) values of χ_TS_, at 25 °C. Error bars correspond to the uncertainty in the fits to the experimental data.

Before we turn to analyze the experimental results in terms of our model, we describe the procedure for determining the necessary model parameters. Cosolute size, given by ν = V̅_C_/V̅_S_, is determined from the partial molar volumes of the cosolute, V̅_C_, and the solvent, V̅_S_. These volumes are determined from density measurements of binary sugar–water mixtures, as described in Section S1.3 in the SI. Solution density values for the sugars used in this study are shown in Figure S1 and Table S6, and the corresponding values of ν are in Figure 4A and Table S2. The similarity in ν values between all monosaccharides as well as between all disaccharides suggests that any difference in native state stability between glucose, galactose, and sorbitol and between trehalose and sucrose must originate from chemical rather than steric interactions.

In addition to the excluded volume interactions, our model considers the contribution of nonideal cosolute–solvent interactions through the parameter χ. These interactions are relevant to protein stability because, due to interactions at the protein interface, protein and bulk domains will generally have different cosolute compositions. Upon protein folding and subsequent cosolute and solvent release, the mixing of the liberated molecules with the differently concentrated bulk generally contributes to the folding free energy.

We determined the values of χ for each cosolute from water activity measurements of binary mixtures, as described in Section S1.4 in the SI. We find χ > 0 for all cosolutes, indicating positive (unfavorable) deviations from a mixture having only excluded volume interactions, Figure 4B and Table S3 in the SI. This contribution represents a net unfavorable nonideal interaction between cosolutes and water. Although χ increases to some extent with the cosolute size, the molecular size alone clearly cannot account for the variability in χ between cosolutes. Importantly, the difference in χ for different carbohydrates suggests that the contribution from solution release is cosolute-specific, even for similarly sized sugars.

The enthalpic and entropic contributions to the nonideal mixing parameter, χ = χ_H_ – χ_TS_, were determined for the water activity of binary cosolute–water mixtures using a van’t Hoff analysis, as detailed in Section S1.4 in the SI. Values of χ_TS_ are shown in Figure 4C, and values of χ_TS_ and χ_H_ at 25 °C are in Table S3 in the SI. We find that χ_TS_ is negative for all cosolutes and that its value varies substantially between sugars. Moreover, we find that except for sorbitol, χ_H_ is negative for all cosolutes, which together with our finding that χ > 0 suggests that the formation of cosolute–solvent contacts incurs an entropic loss.

### Soft Interactions Stabilize MET16 but Destabilize AQ16

Model fits of the experimental ΔΔ*G*^0^, solid lines in Figure 3A,B, use ε as the only fit parameter. Values used for ν and χ are those determined from measurements of binary mixtures in the absence of protein. Model fits succeed in reproducing the diverse ΔΔ*G*^0^ curvatures seen for the smaller and larger sugars. For both miniproteins with the smaller cosolutes composed of six or fewer carbons (i.e., glucose, galactose, sorbitol, and glycerol), ΔΔ*G*^0^ is nearly linear in molality. By contrast, for the larger disaccharides, we find that this dependence is significantly nonlinear.

**Figure 5:**
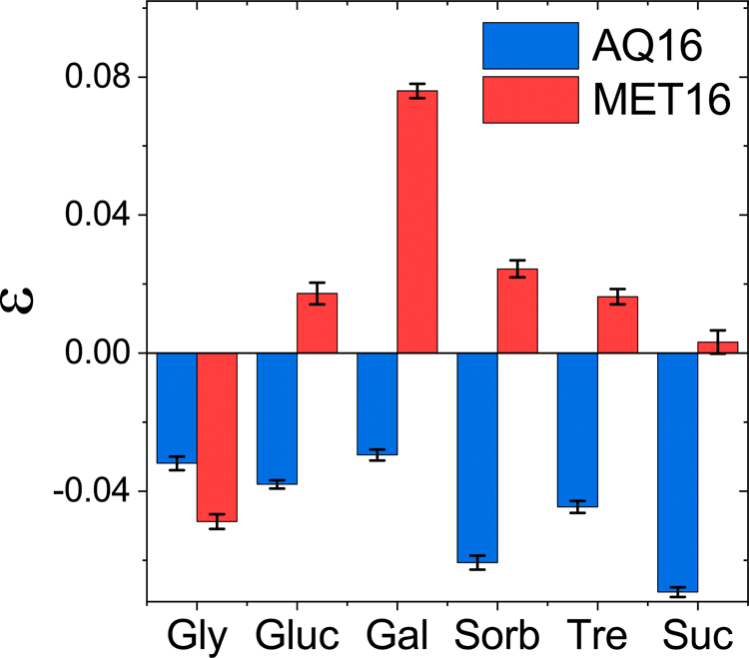
Values of the soft interaction parameter, ε, determined from the model fits of ΔΔ*G*^0^ versus cosolute molality, Figure 3A,B. Error bars correspond to the uncertainty as described in Section S1.6 in the SI.

We find that the sign of ε changes from positive for most sugars with MET16 to negative for all sugars with AQ16, Figure 5 and Table S4 in the SI. Evidently, the same cosolutes show repulsive soft interaction toward MET16’s interface but attractive interaction with AQ16. Stated differently, the cosolute–protein interaction augments the excluded volume steric exclusion with MET16 but counters it with AQ16. The disparity we find in ε values between the miniproteins for the same cosolute demonstrates that the surface chemistry of proteins plays an unexpected yet significant role in the effect of the cosolutes on folding. Interestingly, glycerol is an outlier, with ε being even more negative for MET16 than for AQ16. This relatively strong chemical attraction of glycerol to MET16’s interface explains the rather low stabilization observed in experiment, Figure 3B.

**Figure 6:**
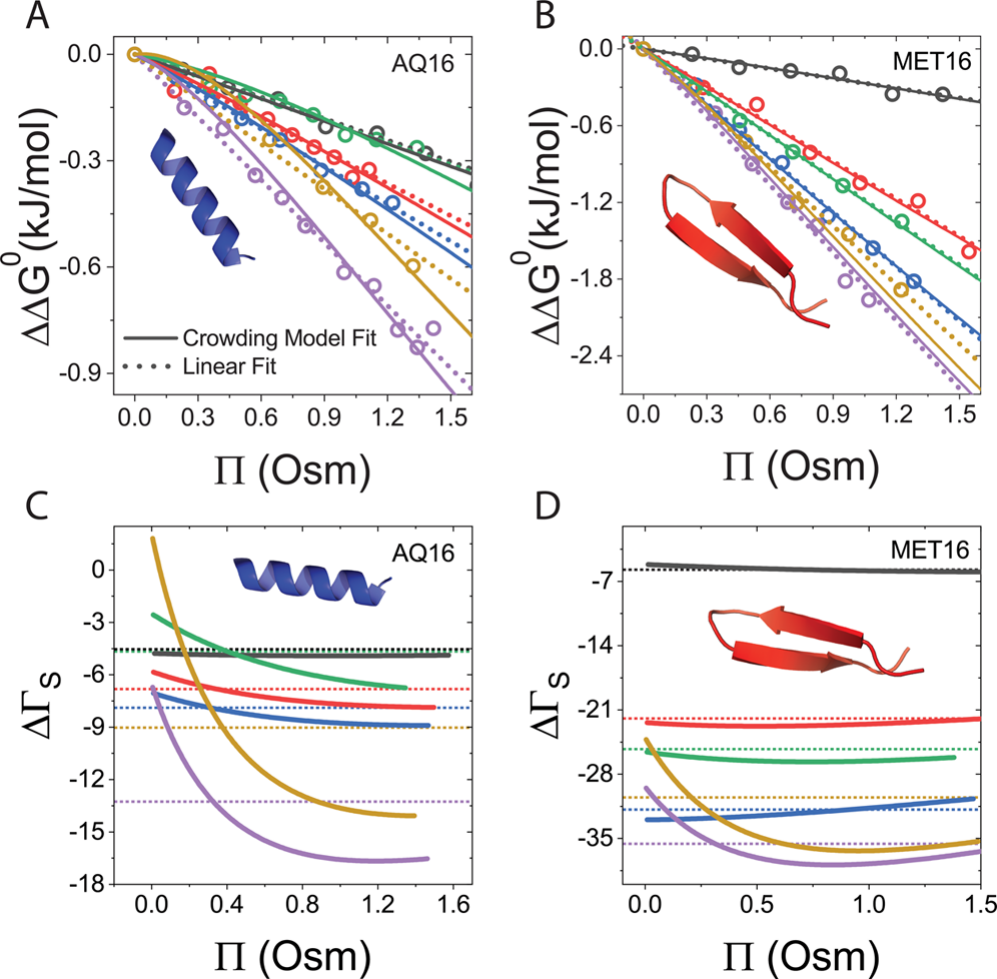
Change in preferential hydration parameter upon protein folding, ΔΓ_S_, in the presence of sugars and polyols. (A) Linear and model fits of AQ16’s ΔΔ*G*^0^ versus cosolutes’ osmotic pressure. (B) Linear and model fits of MET16’s ΔΔ*G*^0^ versus cosolutes’ osmotic pressure. (C) Preferential solvation of cosolutes with AQ16. (D) Preferential solvation of cosolutes with MET16. Full lines are model fits, and dashed lines are linear fits to the data.

Changes in preferential hydration coefficients upon folding, ΔΓ_S_, are derived from the slope of ΔΔ*G*^0^ versus cosolute osmotic pressure, Figure 6A,B.^82−86^ Full curves show fits of the data to our mean-field model, and dashed lines are linear fits commonly used to interpret protein folding data,^50,87,88^ added here to emphasize the significant deviation from linearity of some of the plots. The values of ΔΓ_S_ were determined using the expression
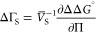
2and shown in Figure 6C,D and Table S5 in the SI for each miniprotein. In eq 2, *V̅*_s_ is the molar volume of the solvent. While ΔΓ_S_ from the linear fits is constant (dotted lines, Figure 6), ΔΓ_S_ from the fits to the mean-field model (solid lines) is concentration-dependent and for larger cosolutes become more negative with increasing Π, which translates to greater sugar exclusion at high osmotic pressure.

### Soft Interactions Determine Specificity of Protein Stabilization by Sugar

**Figure 7:**
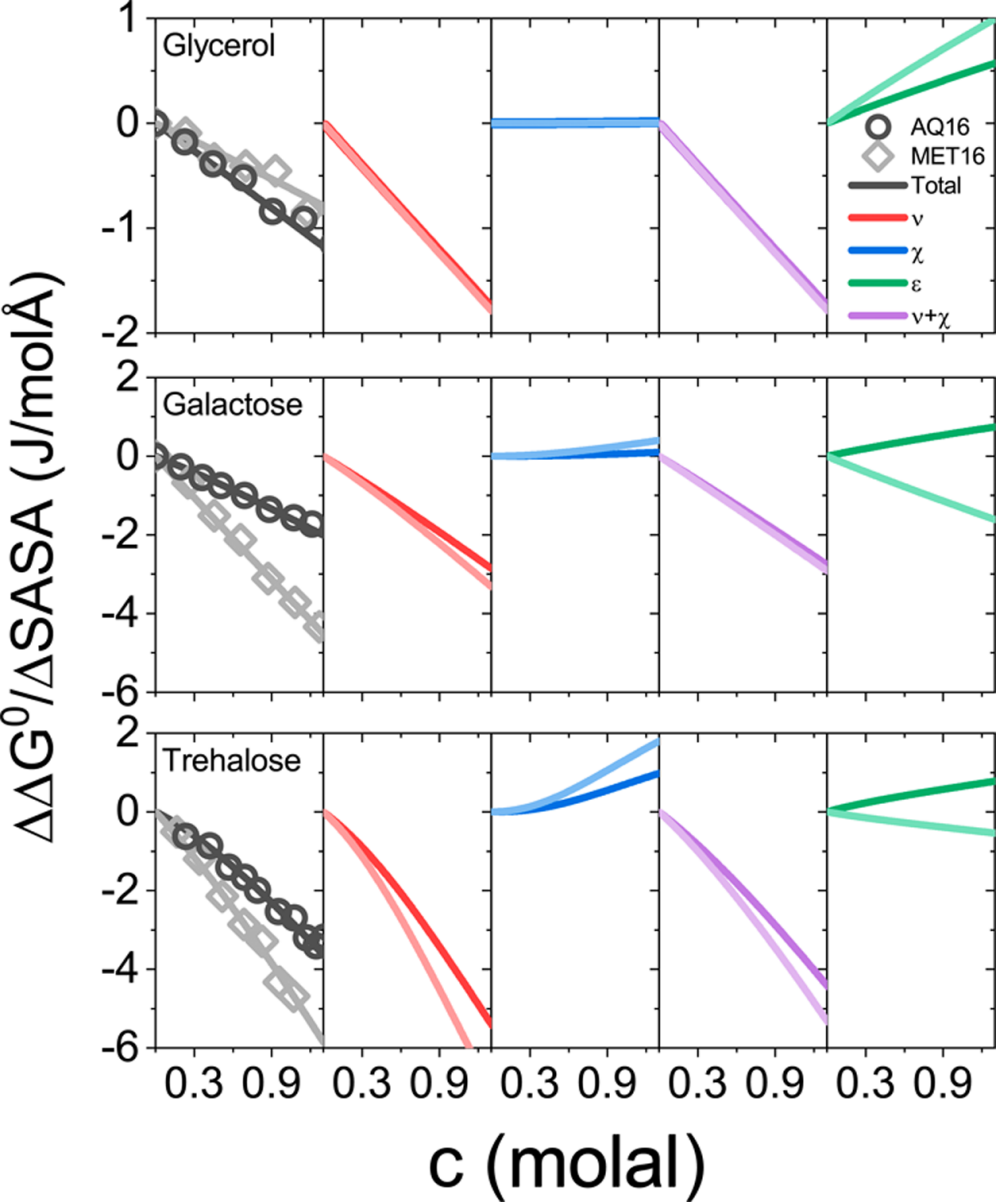
Contributions of ν, χ, and ε to ΔΔ*G*^0^/Δ*SASA* of AQ16 (circles) and MET16 (diamonds) for glycerol, galactose, and trehalose. The total free energies, which are the sum of ΔΔ*G*_ν_^0^, ΔΔ*G*_χ_^0^, and ΔΔ*G*_ε_^0^, are shown in black for AQ16 and gray for MET16.

Our model fits allow us to dissect ΔΔ*G*^0^ into contributions from excluded volume, ΔΔ*G*_ν_^0^, nonideal mixing, ΔΔ*G*_χ_^0^, and soft interactions, ΔΔ*G*_ε_^0^. Figure 7 shows these three contributions to protein stability as well as their sum per change in solvent-accessible surface area upon folding, Δ*SASA*, for three representative cosolutes: glycerol, galactose, and trehalose. Values of Δ*SASA*, Table S1, were determined as described in Section S1.2 in the SI, and free energy contributions for all cosolutes versus cosolute size are given in Figure S6 in the SI.

As expected, the excluded volume is the dominant contribution (red curve) and is stabilizing over the entire concentration range. By contrast, the nonideal mixing contribution (blue curve) is destabilizing. The main contribution that determines the differences in the folding free energy for the two proteins is ΔΔ*G*_ε_^0^ (rightmost panel, Figure 7). We find that this contribution to ΔΔ*G*^0^ is significant for all cosolutes and cannot be neglected. For all cosolutes except glycerol, ΔΔ*G*_ε_^0^ is negative for MET16 but positive for AQ16, resulting in greater stability per surface area for MET16 compared with AQ16 for the same sugar.

Both the stabilizing excluded volume contribution and destabilizing nonideal mixing contribution increase with cosolute size. For example, while ΔΔ*G*_χ_^0^/Δ*SASA* is at least 1 J/molÅ for trehalose concentrations above 1 molal, for glycerol, it is close to zero at all concentrations. The destabilizing contribution of nonideal mixing is a result of the relevant free energy term, ΔΔ*G*_χ_^0^ = χ(*ν*V̅_S_)^2^(*m*^surf^ – *m*)^2^, where *m* and *m*^surf^ are the cosolute molar concentrations in bulk and protein domains, respectively. Specifically, note that ΔΔ*G*_χ_^0^ is quadratic in (*m*^surf^ – *m*), corresponding to the mixing of the differently concentrated bulk and protein domains upon folding. Because χ is positive for all sugars, this nonideal solvent–cosolute mixing term is positive and hence represents an unfavorable, destabilizing contribution to the folding process. Note also that ΔΔ*G*_χ_^0^ necessarily increases with both χ and ν^2^, i.e., the nonideal destabilization increases not only due to stronger nonideal interactions but also because of the larger number of nonideal solvent–cosolute interactions associated with larger cosolutes.

The soft interactions embodied in ε, indirectly influence both ΔΔ*G*_ν_^0^ and ΔΔ*G*_χ_^0^. This is because these terms depend on *m*^surf^ that in turn implicitly depends on all of the model parameters: ν, χ, and ε. The concentration *m*^surf^ is determined from the equilibrium condition, as described in Section S1.2 in the SI. This implicit dependence on ε explains the small difference in ΔΔ*G*_ν_^0^ and ΔΔ*G*_χ_^0^ values per Δ*SASA* for AQ16 and MET16, which naively would be expected to only depend on cosolute identity, and therefore be equal for all proteins, Figure 7. These differences between proteins are more prominent for larger cosolutes because of their greater exclusion, resulting in a larger concentration difference between the bulk and the protein domains.

### Soft Interactions Are Dominated by Enthalpy for MET16 but by Entropy for AQ16

The temperature dependence of the folding free energies allows further dissection of the soft-interaction parameter into enthalpic and entropic components, ε = ε_H_ – ε_TS_. To resolve ε_H_ and ε_TS_, we first determined the folding enthalpy, Δ*H*^0^, and entropy, Δ*S*^0^, by fitting the values of Δ*G*^0^ versus temperature to the integrated form of the van’t Hoff equation,
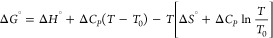
3where the change in heat capacity between the native and denatured states, Δ*C*_*P*_, is assumed to be constant, *T* is the temperature, and *T*_0_ = 298 K is used as the reference temperature. Relaxing the assumption on Δ*C*_P_ did not change the conclusions of the analysis, see Section S1.7 in the SI. The enthalpy and entropy changes due to the addition of sugar, ΔΔ*H*^0^(*c*) = Δ*H*^0^(*c*) – Δ*H*^0^(0) and ΔΔ*S*^0^(*c*) = Δ*S*^0^(*c*) – Δ*S*^0^(0), are determined from fits to eq 3 at different sugar concentrations, as shown for both proteins with the disaccharide trehalose in Figure 3C,D.

**Figure 8:**
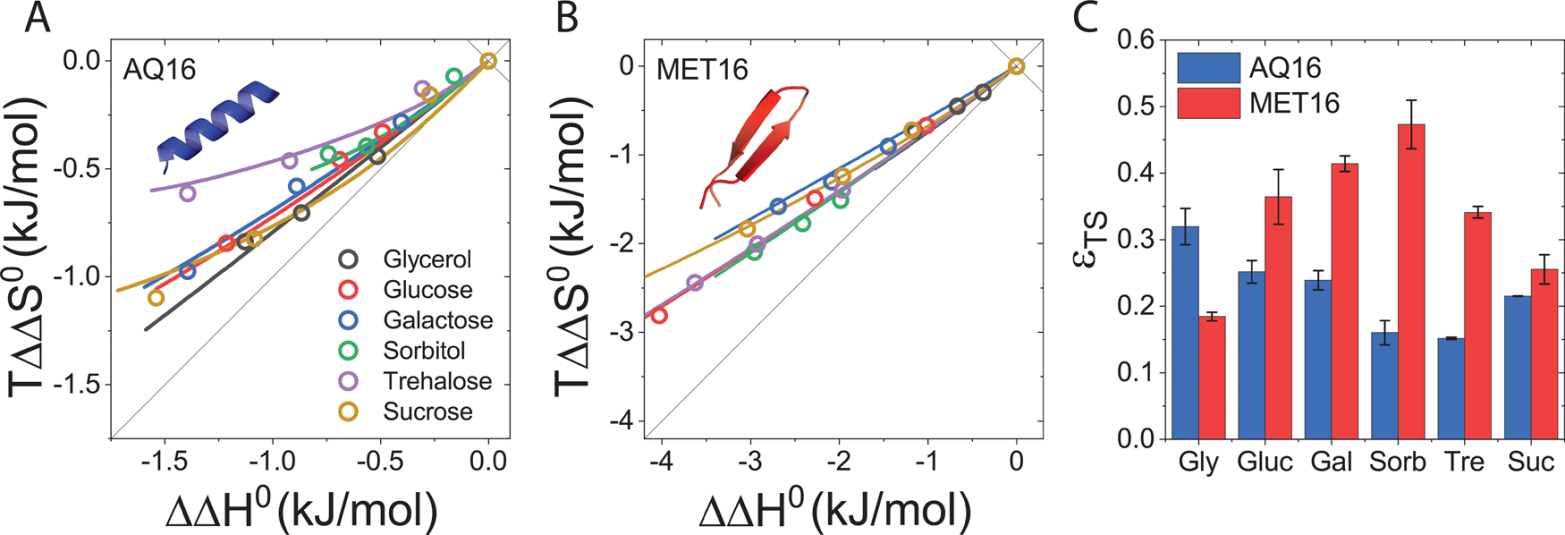
Enthalpic and entropic contributions to folding and soft interactions in the presence of sugars. (A) Folding enthalpy–entropy plot with model fits for AQ16. (B) Folding enthalpy–entropy plot with model fits for MET16. (C) Values of ε_TS_ for the miniproteins. Error bars correspond to the uncertainty as described in Section S1.6 in the SI.

Entropy–enthalpy plots showing *T*ΔΔ*S*^0^ versus ΔΔ*H*^0^ for AQ16 and MET16 are in Figure 8A,B. All cosolutes studied here are stabilizing and are therefore located above the *T*ΔΔ*S*^0^ = ΔΔ*H*^0^ diagonal corresponding to ΔΔ*G*^0^ = 0. Moreover, we find for all cosolutes a large favorable enthalpic contribution, ΔΔ*H*^0^ < 0, partially compensated by a smaller unfavorable entropic contribution, *T*ΔΔ*S*^0^ < 0. This type of enthalpy–entropy compensation, seen here as a strong enthalpic stabilization accompanied by entropic destabilization, is typical for smaller osmolytes, including sugars and polyols.^54^ Larger polymeric crowders usually, but not always,^53^ show a weaker correlation between the enthalpic and entropic contributions.^89^

To resolve the entropic and enthalpic components of the model parameters, we fit the experimental *T*ΔΔ*S*^0^ vs ΔΔ*H*^0^ using the model. Since ε is already determined from fits to ΔΔ*G*^0^, ε_TS_ can be used as the sole fitting parameter in the enthalpy–entropy fits. Thus, the two parameters, ε and ε_TS_, allow us to fit not only the folding free energy but also its enthalpic and entropic contributions. Fits for all cosolutes, Figure 8A,B is in good agreement with the experimental data, within measurement error.

The soft interactions of cosolute with the protein are determined by a balance of entropic attractions and enthalpic repulsions. Figure 8C shows the values of ε_*TS*_ obtained from these fits. We find that ε_TS_ is positive for all protein–cosolute pairs. Likewise, ε_H_ is also invariably positive (Figure S8 and Table S4 in the SI). The positive sign of ε_TS_ means that the formation of protein–cosolute contacts leads to an entropic gain that favors cosolute inclusion. The positive sign of ε_H_ translates to protein–cosolute contacts that are energetically weaker than protein–water contacts and therefore contributes to the exclusion of cosolute from the protein. The entropic gain upon formation of protein–cosolute contacts is consistent with a water release mechanism, in which water molecules liberated from the protein interface gain accessible states or degrees of freedom. The concomitant reduction in enthalpy may indicate that favorable protein–water Hbonds are lost as water is released, and these are only partially compensated by weaker protein–cosolute hydrogen bonds.^90^ We return to discuss the link between soft interactions and hydrogen bonding in the following sections.

While for AQ16 entropic cosolute–protein attractions dominate, for MET16 enthalpic repulsions largely prevail. For both proteins, the large (compensating) values of ε_H_ and ε_TS_ compared to ε, result in large ΔΔ*H*_ε_^0^ and *T*ΔΔ*S*_ε_^0^ contributions. These contributions account for the strong enthalpy–entropy compensation that we find see Figure S7 in the SI. Further details on the enthalpic and entropic contributions to ΔΔ*G*_ν_^0^, ΔΔ*G*_χ_^0^, and ΔΔ*G*_ε_^0^ are given in Section S7 in the SI.

### Simulations Reveal Sugar Exclusion from MET16 but Inclusion around AQ16

To gain molecular-level insight into the distinct soft interactions of cosolutes with the two miniproteins, we performed molecular dynamics (MD) simulations of AQ16 and MET16 in pure water and in 1.3 molal of the disaccharides trehalose and sucrose at 25 °C. AQ16 and MET16 were simulated starting both in the native and denatured states. Our strategy was to exploit the faster relaxation times of sugar and water mixing compared to the slower folding times of the miniproteins to study the structure of the solution around each state separately, thereby allowing us to determine cosolute preferential interaction coefficients.^91^ In all simulations, we employed the modified force field CHARMM36 developed by Cloutier et al.,^92^ together with the TIP3P water model,^93^ using the GROMACS package.^94^ Simulation details, validation of the applied force field, and method for determining preferential interaction coefficients are listed in Section S9 in the SI.

We find that for MET16 in the simulations, Γ_S,N_ is close to zero but Γ_S,D_ is positive. These values are in qualitative agreement with our previously reported osmotic pressure measurements showing the exclusion of trehalose from the interface of MET16.^81^ In contrast, sugars are included (Γ_S_ < 0) near both the native and denatured states of AQ16. The negative sign of ΔΓ_S_ that is observed in the folding experiments of both proteins is also seen in the simulations since Γ_S,N_ is invariably smaller than Γ_S,D_, Figure S10. The difference in sign for Γ_S_ for the two proteins necessarily reflects different protein–cosolute soft interactions of sugars with AQ16 compared to MET16. Specifically, the attraction of sugar to AQ16 (ε < 0) and repulsion from MET16 (ε > 0) is supported by the preferential inclusion and exclusion seen in the simulations. Overall, we find that the changes in preferential hydration coefficients upon folding in the simulations are somewhat larger but in good qualitative agreement with the experimental results, Section S9 in the SI. In the following section, we further analyze these simulations to shed light on the molecular origins of the differences in soft interactions between sugar and the two proteins.

### Hydrogen Bonding Stabilizes MET16 but Destabilizes AQ16

To resolve the dominant force that leads to the marked differences in the interaction of sugars with the two proteins, we calculated the contributions of van der Waals and electrostatic interactions to the change in potential energy for folding due to the addition of sugar, ΔΔ*E*_vdW_ and ΔΔ*E*_el_. For both proteins, the absolute value of ΔΔ*E*_vdW_ is at least 1 order of magnitude smaller than that of ΔΔ*E*_el_, Table 1. Moreover, ΔΔ*E*_el_ is negative for MET16 but positive for AQ16, while the signs of ΔΔ*E*_vdW_ vary greatly, suggesting that the added stabilization by sugar soft interactions for MET16 and the destabilization for AQ16 originate in electrostatic rather than van der Waals interactions. Importantly, hydrogen-bonding interactions are mostly related to the electrostatic potential part of the empirical force fields, indicating that hydrogen bonds may potentially play a significant role in modulating native state stability.

**Table 1:** Difference in Energy Between Native and Denatured Protein States due to the Addition of Sugar

		ΔΔ*E*_el_ (kJ/mol)	ΔΔ*E*_vdW_ (kJ/mol)	ΔΔ*E*_nb_^a^ (kJ/mol)	*P*ΔΔ*V* (kJ/mol)	ΔΔ*E*_tot_^b^ (kJ/mol)
MET16	Tre	–679.18	–1.57	–680.75	0.0011	–6.16
	Suc	–418.91	8.75	–410.17	0.0009	–39.37
AQ16	Tre	384.65	–31.36	353.29	0.0034	–37.93
	Suc	1166.77	4.06	1161.83	–0.0015	–49.89

a Sum of nonbonded potential energies, ΔΔ*E*_nb_ = ΔΔ*E*_el_ + ΔΔ*E*_vdW_.

b Total energy, sum of potential and kinetic energies.

To test the possible role of hydrogen bonding in the cosolute–protein interactions, we applied our information-theory-based methodology to calculate the strength of protein–sugar and protein–water hydrogen bonds (Hbonds). The methodology allows us to assign a free energy to a single Hbond, denoted Δ*G̅*_i_^N/D^, that is unique to a donor–acceptor pair, *i*, and for a protein state, N or D. This Hbond free energy is determined from the probability density of finding in simulations the Hbonded pair at any specific distance and relative angles. Details on the methodology are given in refs (95) and (96) and described in Section S10 in the SI. A total Hbond network free energy can be defined by summing over all Hbond free energies in the system. Then, the change in total Hbond free energy for the native or denatured states upon sugar addition is
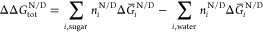
4where the *n*_*i*_^N/D^’s are the average number of Hbonds between pairs of type *i*, and the sums are taken over Hbond pairs in the presence and absence of sugar. Finally, the change in total Hbond free energy upon folding due to the addition of sugar is ΔΔ*G*_fold_ = ΔΔ*G*_tot_^N^ – ΔΔ*G*_tot_^D^.

**Figure 9:**
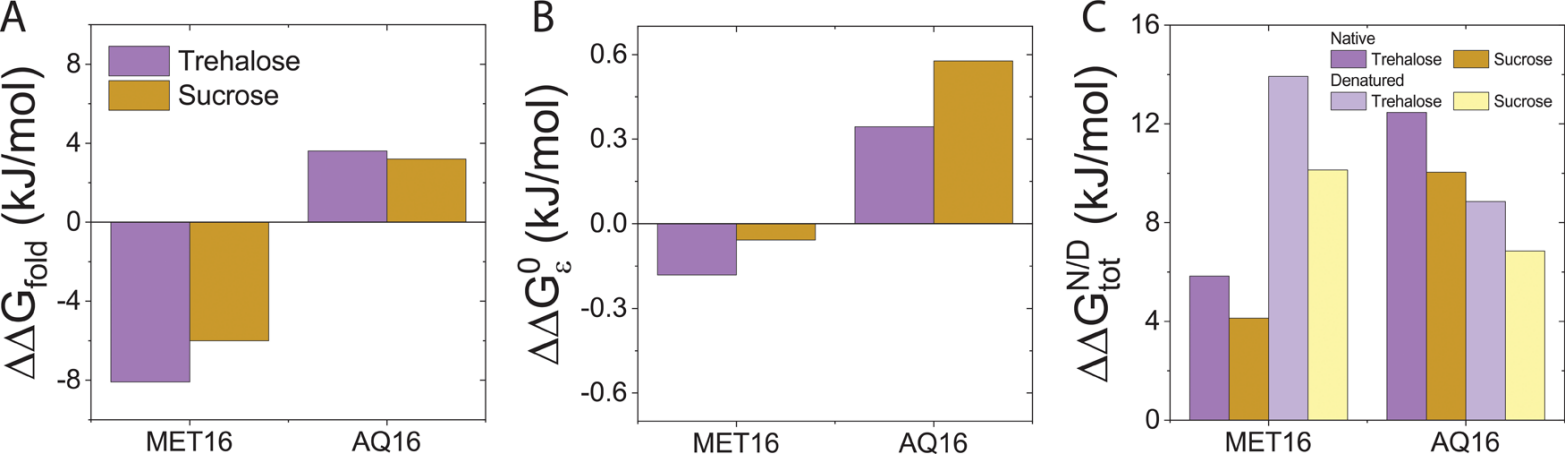
Effect of added sugars on protein’s Hbond free energy. (A) Change in Hbond contribution to folding free energy due to added sugar. (B) Soft interactions contribution to the folding free energy at *c* = 1*molal*. (C) Change in native and denatured states Hbond free energy upon sugar addition.

Figure 9A shows ΔΔ*G*_fold_ for MET16 and AQ16. We find that the Hbond contribution to protein stability due to added sugar is stabilizing for MET16 but destabilizing for AQ16, in accordance with the soft interaction contributions we resolve from experiments, Figure 9B. For MET16, changes in Hbonding can also account for the greater stabilization by trehalose compared to sucrose seen in folding experiments, but for AQ16, the Hbond’s effect of trehalose and sucrose on protein stability is comparable, while trehalose is more stabilizing than sucrose in experiments. These findings suggest that protein–solution Hbonds likely play an important role in determining the emerging soft interactions.

To discern the differences that result in Hbond stabilization of MET16 but destabilization of AQ16 by Hbonds, we follow the change in Hbond free energy for the native and denatured states ΔΔ*G*_tot_^N/D^, Figure 9C. The native and denatured states of both proteins are destabilized by added sugars, seen as positive values of ΔΔ*G*_tot_^N^ and ΔΔ*G*_tot_^D^. However, while for MET16, the denatured state is more destabilized than the native state (ΔΔ*G*_tot_^D^ > ΔΔ*G*_*tot*_^*N*^), for AQ16, the native state is more strongly destabilized.

**Figure 10:**
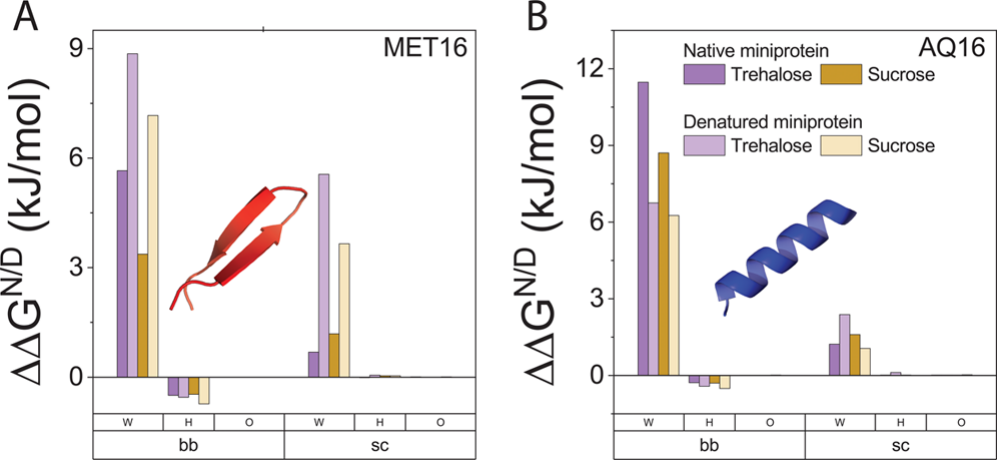
Protein backbone (bb) and side chains (sc) Hbond interactions with water (W), sugar hydroxyl (H), and sugar ether (O). (A) Change in protein–water and protein–sugar Hbond free energy in the presence of sugars for MET16. (B) Change in protein–water and protein–sugar Hbond free energy in the presence of sugars for AQ16.

Further dissection into individual contributions of protein Hbonds with water, sugar hydroxyls, and sugar ethers reveals that the destabilization of the native and denatured states by added sugar originates in the less favorable protein–water Hbonding in the presence of sugar relative to Hbonds in pure water, Figure 10A,B. By contrast, the contribution of protein Hbonds with sugar hydroxyls is small, and with sugar ethers it is negligible. The contribution of interactions with individual side chains that determine the details of specificity of Hbond interactions due to added sugar is discussed in Section S10 in the SI.

### Hydrogen Bond Strength but Not Their Number Determine Protein Stability

**Figure 11:**
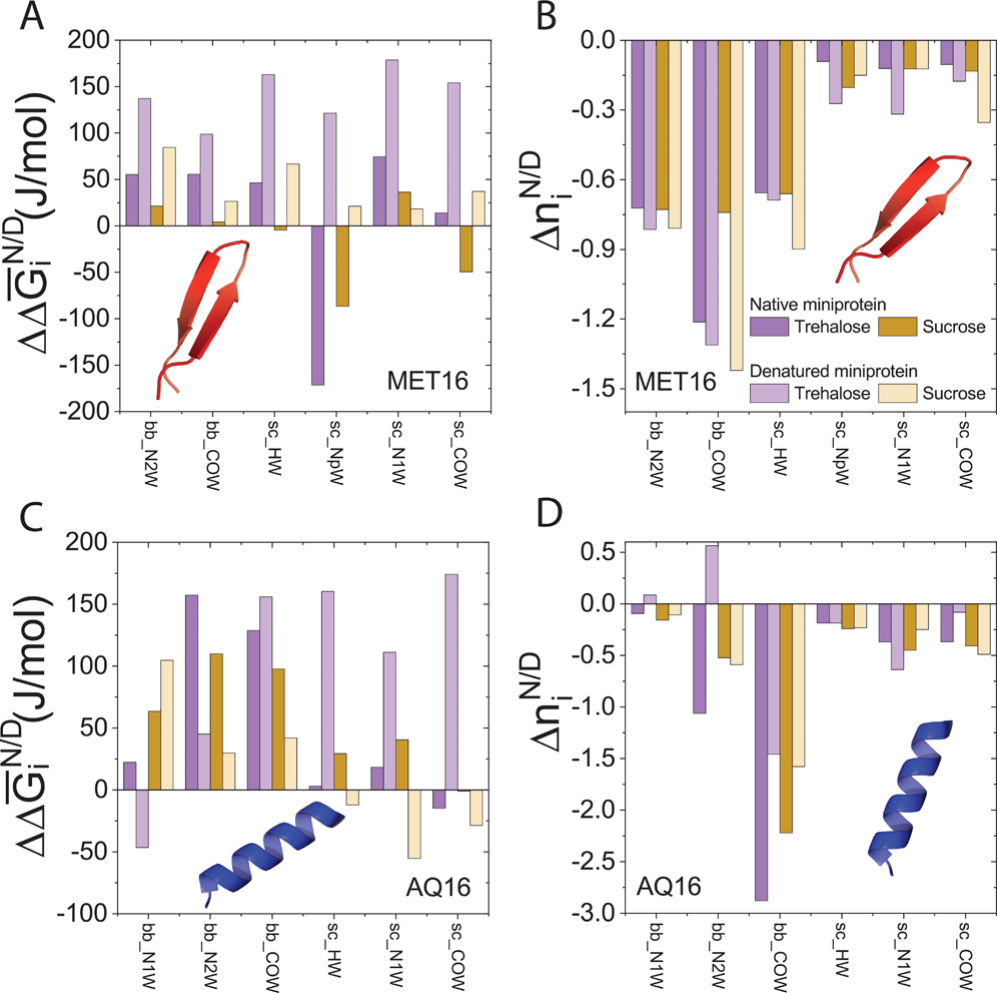
Change in protein–water Hbond strength and number of Hbonds upon sugar addition. MET16’s change in Hbond (A) strength and (B) number. AQ16’s change in Hbond (C) strength and (D) number.

So far, we have shown that the clear effect of sugar on protein–water hydrogen bonding accounts for the difference between the impact of the same sugar (trehalose or sucrose) on AQ16 versus MET16. We now show that sugars impact protein stability by modifying the strength of protein–water Hbonds rather than their number. Figure 11 shows the change in protein–water Hbond strength, ΔΔ*G̅*_*i*_^N/D^, and the change in number of Hbonds, Δ*n*_*i*_^N/D^, due to sugar addition. For MET16, the protein–water Hbonds around the denatured state weaken more than those around the native state, resulting in the stabilization of the native state seen in Figure 9. However, for AQ16, the backbone–water Hbonds weaken more around the native state than the denatured state, resulting in destabilization of the native state. The effect of added sugar on AQ16’s side chains Hbonds is negligible, Figure 10B. By contrast, the number of protein–water Hbonds generally decreases in the presence of sugar molecules, by 1.4 Hbonds for MET16 and by 2.9 Hbonds for AQ16. This systematic loss in the number of Hbonds contributes to the increase in ΔΔ*G*_tot_^N^ and ΔΔ*G*_tot_^D^ seen in Figures 9 and 10. However, since Δ*n*_*i*_^N/D^ is similar for the native and denatured states of each miniprotein, Δ*n*_i_^N/D^, cannot account for the stabilization of MET16 versus destabilization of AQ16. Taken together, our simulations suggest that added sugar interferes with interactions of proteins with their surroundings by weakening the protein–water Hbond strength. For which state, native or denatured, is weakened more depends sensitively on the protein and the interface it presents to the surrounding solution.

### Relating Hydrogen Bonding and Protein Structure to ε

Matching specific interactions in atomistic simulations with the empirical parameters from mean-field theory generally poses a challenge because of the very different frameworks in which each is set. Nevertheless, some correlations are apparent: while our model reveals that soft interactions are significant and determine the specificity of protein stabilization by sugar, Figure 7, the simulations suggest that Hbonds play a crucial role in this specificity, Table. 1. In addition, we find that the model’s soft interaction contributions and the influence of sugars on protein Hbonds within simulation are well correlated. Specifically, the signs of ΔΔ*G*_fold_ and ΔΔ*G*_ε_^0^ match for both proteins: negative for MET16 and positive for AQ16, Figure 9A, B. Moreover, our simulations show that ΔΔ*G*_fold_ results from weakening of protein–water Hbonds that are not fully compensated by the formation of protein–sugar Hbonds. These findings suggest a strong contribution of Hbonds to the soft interactions as defined in our model parameter ε. In particular, a greater weakening of protein–water interactions for the denatured state compared to that for the native state in the presence of sugar results in a positive ε, while greater weakening for the native state corresponds to a negative ε.

Why is the native state of AQ16 more destabilized upon sugar addition than the denatured state, while the reverse is true for MET16? For AQ16, which folds into an α-helix through an exothermic mechanism, we find that the native and denatured states are both preferentially dehydrated in the presence of sugar, Figure S10B. The reduced hydration of the protein has two consequences: (i) a decline in the number of protein–water hydrogen bonds and (ii) increased frustration among water molecules because of the unavoidable disruption of the Hbond network due to the more rigid sugar molecules. Because the native state of AQ16 shows even stronger dehydration compared to the denatured state, it undergoes a more significant decrease in protein–water hydrogen bonds as well as a concurrent weakening of each individual hydrogen bond.

Conversely, MET16 folds into a β-hairpin driven by entropy, suggesting hydrophobic interactions are an important driving force in folding. For MET16, we observe that the denatured state is further hydrated in the presence of sugar, but there is no discernible impact on the hydration of the native state, Figure S10A. In our simulations, the water molecules surrounding MET16 in the presence of sugar exhibit stronger hydrophilic characteristics compared to pure water. Specifically, we find that in this environment water molecules further prioritize the preservation of water–water hydrogen bonds over the formation of weaker hydrogen bonds with the protein. Indeed, we have previously demonstrated that the strength of water–water Hbonds increases in the presence of sugars.^97^ Due to the higher hydration level in the denatured state compared to the native state of MET16, the protein–water Hbonds experience a more significant penalty in the denatured state. This trend contributes to an overall increase in the folding stability of MET16. Extending and generalizing our conclusions from this analysis of only two model proteins, each exhibiting distinct folds, to larger globular proteins containing multiple secondary structural elements warrants further investigation.

## Conclusions

Most studies of protein folding in the presence of added cosolutes only consider excluded volume effects and neglect chemical interactions between the cosolute, protein, and solvent. Models that do include additional protein–cosolute interactions usually assume that they are attractive, and furthermore do not consider nonideal mixing terms for the solution. Here, we implemented a mean-field model that is based on FH theory to fit and dissect the folding thermodynamics of two model miniproteins in the presence of different polyols and sugars. Importantly, our model includes contributions from excluded volume and soft protein–cosolute interactions (that can be attractive or repulsive) as well as cosolute–solvent nonideal mixing terms.

We followed the reversible folding of two miniproteins with temperature and carbohydrate cosolute concentration by using CD spectroscopy. By fitting our model to the folding ΔΔ*G*^0^, ΔΔ*H*^0^, and *T*ΔΔ*S*^0^, we have simultaneously resolved the nonideal mixing and soft interaction contributions to protein folding using only a small set of model parameters.

We find that the soft interactions of all cosolutes with AQ16’s exposed interface are net attractive but are repulsive for most cosolutes with MET16. Protein–cosolute attractions diminish the stabilizing effect of excluded volume interactions on AQ16, while repulsions further stabilize MET16. For both miniproteins and for all cosolutes, the soft interactions comprise an entropic attraction and almost fully compensating enthalpic repulsion, suggesting a weakening of protein interactions with the solvating solution in the presence of sugar.

Molecular dynamics simulations allowed us to further probe the origins of the disparate soft interactions with the two miniproteins. We show that protein–cosolute soft interactions are intimately related to Hbonds that the miniproteins form with the solution. Specifically, we find that upon addition of sugar, Hbonds stabilize MET16 but destabilize AQ16. This effect on protein stability originates from the unequal weakening of Hbonds formed between the native and denatured states with their solvating water molecules. In other words, the soft protein–cosolute interaction, which can enhance or reduce protein stability, originates mainly from sugar-induced changes in the structure of water in the vicinity of the native and denatured states. The restructuring of water molecules at the protein interface and subsequent changes in Hbond strength are sensitive not only to cosolute concentration but also to sugar and protein identities. It is this difference in Hbond interactions that ultimately determines the specific impact of added sugars on the stability of different proteins, even for the same sugar. This allows the emergence of specific interactions, even though the change in folding free energy due to sugar is, in most cases, dominated by the sugar excluded volume, which depends only on sugar size. Combining our mean-field model with MD simulations has allowed us to bridge the gap between the thermodynamic and molecular-level mechanisms of carbohydrate-specific impact on protein stability.

## Supporting Information Available

Materials and methods; model parameters; uncertainties in ε; analysis of Δ*C*_*P*_; aqueous mixtures densities, water activities, and osmotic pressures; AQ16 spectra in the presence of trehalose; contributions to ΔΔ*G*^0^, ΔΔ*H*^0^, and *T*ΔΔ*S*^0^; force field validation; preferential hydration parameters; and Hbond methodology (PDF)
