# Supplementary material for: Not Always Sticky: Specificity of Protein Stabilization by Sugars Is Conferred by Protein–Water Hydrogen Bonds
Source: J Am Chem Soc. 2023 Oct 16;145(42):23308–20. doi: 10.1021/jacs.3c08702 (PMC10603812; doi:10.1021/jacs.3c08702)
Supplement: Supplementary file 1 — ja3c08702_si_001.pdf [file ja3c08702_si_001.pdf]

Supporting Information: Not always sticky: Specificity of Protein Stabilization by Sugars is Conferred by Protein-Water Hydrogen Bonds

Gil I. Olgenblum, Neta Carmon, and Daniel Harries*

Institute of Chemistry, the Fritz Haber Research Center and the Harvey M. Kruger Center for Nanoscience & Nanotechnology, The Hebrew University, Jerusalem 9190401, Israel;

E-mail: daniel.harries@mail.huji.ac.il

S1. Materials and Methods

S1.1. Materials

Lyophilized AQ16 and MET16, with sequences Ac-AAQAAAAQAAAAQAA-NH_2 and Ac-KKYTVSINGKKITVSI, at 98% purity were from GenScript Biotech Corporation and kept at −20°C until use. Glycerol (≥99.5%), D-glucose (≥99.5%), D-galactose (≥99%), and sucrose (≥99.5%) were from Sigma-Aldrich, Merck. D-sorbitol (≥99.5%) was from Fluka and trehalose dihydrate (≥99%) was a gift of Hayashibara. All sugars and polyols were used without further purifications. Aqueous cosolute solutions were prepared gravimetrically (Sartorius CP225D Analytical Balance) with previously purified (Barnstead Nanopure Diamond water purification system) and boiled water.

S1.2. Crowding Model

Our model is based on the Flory-Huggins (FH) theory for binary solutions that accounts for the effect of the difference in solvent and cosolute molecular size on the mixing entropy,

as well as non-ideal mixing terms.[S1,S2] The main advantage of using the FH theory is that the chemical potentials of all solution components can be expressed using a small set of parameters, specifically the excluded volume parameter, ν, the non-ideal mixing parameter, χ, and its non-ideal enthalpic and entropic contributions $\chi = \chi_H - \chi_{TS}$.

The FH mixing free energy, Eq. 1 of the main text, is:

$$\frac{\Delta G}{kT} = M_0 \left[\phi_S \ln \phi_S + \frac{\phi_C}{\nu} \ln \phi_C + \chi \phi_S \phi_C \right] \tag{S1}$$

where ϕ_S and ϕ_C are the solvent and cosolute volume fractions, $M_0 = (N_S + \nu N_C)$ is the total volume of solution (expressed in terms of number of lattice sites that solvent and cosolute inhabit), k is the Boltzmann constant, and T is temperature.

ΔG can be dissected into an enthalpic and entropic contributions:

$$\Delta H = kTM_0 \chi_H \phi_S \phi_C \tag{S2}$$

$$\Delta S = -kM_0(\phi_S \ln \phi_S + \phi_C \ln \phi_C - \chi_{TS} \phi_S \phi_C) \tag{S3}$$

and the solvent and cosolute chemical potentials, μ_S and μ_C, can be derived as

$$\mu_{S/C} = \left(\frac{\partial \Delta G}{\partial N_{S/C}} \right)_{P,T,N_{C/S}} \tag{S4}$$

where P is pressure.

We further extend the FH theory to ternary mixtures (cosolute, water, and protein) by dividing the mixture into two domains: the protein and bulk domains. The protein domain is defined as the volume in the vicinity of the protein surface that is buried upon folding, and the bulk spans the part of the mixture unperturbed by the protein. Specifically, the surface area of the protein domain corresponds to the change in solvent accessible surface areas upon folding, $\Delta SASA = SASA_N - SASA_D$, and the extent of the protein domain into solution is defined by the length scale set by the cosolute, $a = \nu^{1/3}$. The values of $\Delta SASA$ for both proteins (MET16 and AQ16) were calculated using the ProtSa server[S3,S4] and given in Table S1.

In our model, protein folding corresponds to the burial of a section of the protein surface, translating to the removal of some of the solvent accesible surface from contact with the mixture. This surface removal releases cosolute and solvent molecules that were previously confined to the protein domain into the bulk. Therefore, the folding free energy can be calculated from the difference between the mixing free energies with and without the removed protein surface in the mixture. The mixing free energy excluding the buried surface is given by the standard FH expression, Eq. 1, whereas the mixing free energy with the surface is

comprised of the FH and an additional protein domain mixing free energy expressions.

For the protein domain, we modify the mixing free energy compared to the bulk in two ways. First, the mixing volume in the protein domain is reduced from $M_{\rm surf}$ to $M_{\rm mix}$ on account of the cosolute excluded volume that restricts the number of available lattice sites. Consequently, we rescale the volume fractions in the protein domain,

$$\begin{aligned}\phi_C^{\rm mix} &= \phi_C^{\rm surf}\left[1-\frac{1}{2}\left(1-\frac{1}{a}\right)\left(1-\phi_C^{\rm surf}\right)\right]^{-1} \\ \phi_S^{\rm mix} &= 1-\phi_C^{\rm mix}\end{aligned} \tag{S5}$$

where $\phi_{S/C}^{\rm mix}$ and $\phi_{S/C}^{\rm surf}$ are the rescaled and unscaled protein-domain volume fractions, respectively. Second, we add a soft interaction term between the cosolute and the protein surface. This term is expressed using the parameter ε, that describes the effective protein-cosolute interaction per exposed protein surface area in terms of free energy. We note that this constant is similar to the interaction term also introduced by Schellman.[S5] These modifications to the free energy result in the following mixing free energy associated with the protein domain:

$$\frac{\Delta G}{kT} = M_{\rm mix}\left(\phi_S^{\rm mix}\ln\phi_S^{\rm mix} + \frac{1}{\nu}\phi_C^{\rm mix}\ln\phi_C^{\rm mix}\right) + \chi M_{\rm surf}\phi_S^{\rm surf}\phi_C^{\rm surf} + \frac{\varepsilon}{a}\phi_C^{\rm surf} \tag{S6}$$

The contribution from the term containing ε can be either stabilizing or destabilizing. Specifically, repulsive soft-interactions, $\varepsilon > 0$, result in a stabilizing contribution to the protein's native state, whereas attractive soft-interactions, $\varepsilon < 0$, result in a destabilizing contribution. Moreover, like χ, ε can in principle depend on temperature, with corresponding enthalpic and entropic terms, $\varepsilon = \varepsilon_H - \varepsilon_{TS}$. We have previously shown that this temperature dependence of the soft interaction is necessary to fully describe an enthalpically stabilizing and entropically destabilizing cosolute.[S6]

Eq. S6 is useful only as long as the volume fractions of solvent and cosolute in the protein domain are known. To determine $\phi_{S/C}^{\rm surf}$, we numerically solve the equilibrium condition:

$$\nu\mu_S^{\rm bulk} - \mu_C^{\rm bulk} + \mu_C^{\rm surf} - \nu\mu_S^{\rm surf} = 0 \tag{S7}$$

where $\mu_{S/C}^{\rm bulk}$ are the bulk chemical potentials and $\mu_{S/C}^{\rm surf}$ are the chemical potentials in the protein domain. The chemical potentials are calculated using Eq. S4 with Eq. 1 for the bulk or with Eq. S6 for the protein domain. Eq. S7 is solved with respect to ε for a specific set of $\Delta SASA$, ν, and χ.

S1.3. Density Measurements and Determination of ν

Values for the excluded volume parameter, ν, were determined from density measurements of binary solutions. For each cosolute, the density of solutions for at least seven different cosolute concentrations were measured using a DMA 5000 M (Anton Paar) densitometer at 25°C. Each cosolute's concentration ranged from 0 (pure water) to near saturation, or up to $\sim$ 4.8 molal, the lower of the two. The measured densities as a function of cosolute concentration are in Fig. S1A and Table S6.

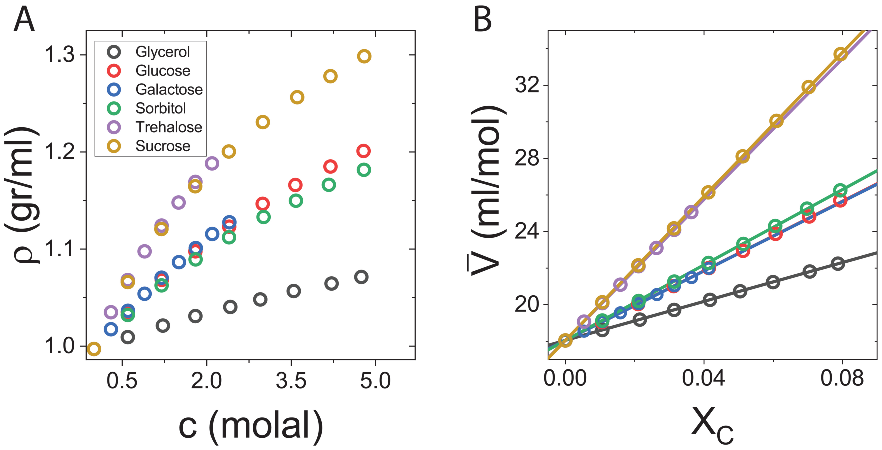

Figure S1: Determination of cosolute partial molar volume. **(A)** Measured densities of aqueous cosolute mixtures at $25°C$. **(B)** Molar volume of binary solutions, calculated using Eq. S8. Solid lines show linear fits to the data.

The molar volume of the mixture, $\bar{V}$, is defined as

$$\bar{V} = \frac{1}{\rho} \left(X_C M_C + X_S M_S \right) \tag{S8}$$

Where ρ is the density in units of gr/ml, X_S and X_C are the solvent and cosolute molar fractions, and M_S and M_C are the solvent and cosolute molar masses.

Fig. 4B shows the molar volumes for different mixtures as a function of the cosolute molar fraction, X_C. The linear relation that the data shows for $\bar{V}$ versus X_C indicates that mixture molar volume is a linear combination of concentration-independent cosolute and solvent partial molar volumes, $\bar{V}_C$ and $\bar{V}_S$, so that $\bar{V} = \bar{V}_S + X_C \left(\bar{V}_C - \bar{V}_S \right)$. The values of $\bar{V}_S$ and $\bar{V}_C$ can then be determined for each cosolute by extrapolating to infinite dilution or to the pure cosolute limits:

$$\bar{V}_S = \lim_{X_C \to 0} \bar{V}$$

$$\bar{V}_C = \lim_{X_C \to 1} \bar{V}$$

Then, ν is calculated as the ratio of the solvent and cosolute partial molar volumes,

$$\nu = \frac{\bar{V}_C}{\bar{V}_S}$$

S1.4. Water Activity Measurements and Determination of χ

The non-ideal interaction parameter, χ, for each cosolute is determined from measurements of water activity, a_S. Water activities of aqueous cosolute mixtures were measured at different temperatures using an AQUALAB 4TE activity meter as follows. The activity meter inner temperature controller was initially set to either 15, 20, 25, or 45°C. Once the sample chamber temperature had settled, the activity meter offset was calibrated using standard salt solutions. For each measurement, ∼3ml of aqueous cosolute mixture was loaded into a measurement plate that was then inserted into the sample chamber and the chamber was closed. After the sample temperature was allowed to equilibrate, the activity was measured. Each reported value represents and average over at least three repeats, Tables S7 – S12.

The measured water activities were converted to osmotic pressure using

$$\Pi = -\frac{RT}{\bar{V}_S} \ln a_S \tag{S9}$$

χ was then determined from fits to the FH expression for the osmotic pressure, Π:[S7]

$$\frac{\Pi \bar{V}_S}{kT} = -\left[\ln \phi_S + \phi_C \left(1 - \frac{1}{\nu}\right) + \chi \phi_C^2\right] \tag{S10}$$

Fig. S2A shows the FH fits to the osmotic pressure as function of volume fraction for each cosolute at $25°C$.

In addition, in order to verify the accuracy of our measurements, we also measured Π as a function of ϕ_C, on an APRO 5520 Wescor osmometer at ambient temperature (with the room temperature set to 25°C), after calibration using standard salt solutions. The measured osmotic pressures are shown in the insets of Fig. S4 and in Table S13. Each data point represents the average of at least three repeats.

Finally, we further dissected χ into its enthalpic, χ_H, and entropic, χ_{TS}, contributions, as follows. First, we determined the values of χ for several temperatures as described above, see fits in Fig. S4. Then, the values of χ as function of T for each cosolute were fitted to either a linear function or to the following Padé approximant:

$$\chi = \frac{b_0 + b_1 T^{-1}}{1 + b_2 T^{-1}}$$

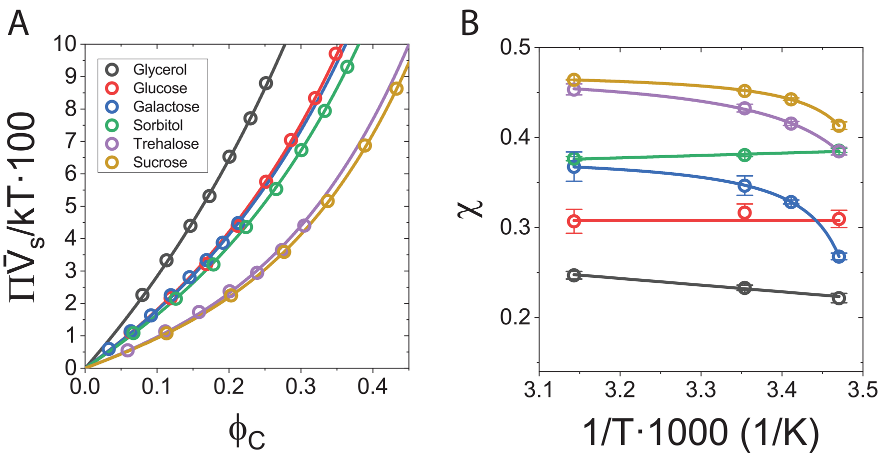

Figure S2: Determination of the FH non-ideal mixing parameter, χ. **(A)** Fits to the FH scaled osmotic pressure, Eq. S10. **(B)** Van 't Hoff analysis of the non-ideal mixing parameter, χ.

These fits, shown in Fig S2B, were used to calculate χ_H by using the Van 't Hoff relation:

$$\left(\frac{\partial\chi}{\partial T^{-1}}\right)_P = T\cdot\chi_H$$

The value of χ_{TS} at 25°C was then calculated as $\chi_{TS} = \chi_H - \chi$. Values of χ and χ_{TS} are plotted in Fig. 4B, C and given in Table S3.

S1.5. Circular Dichroism Measurements and Determination of ε

Circular dichroism (CD) spectra of AQ16 and MET16 in aqueous solutions were measured for a range of cosolute concentrations and temperatures using a J-810 spectrophotometer (JASCO, Japan). Aqueous samples containing either $\sim 50\mu$M AQ16 or $\sim 100\mu$M MET16 were prepared gravimetrically from lyophilized powders and the protein concentrations were determined by following the absorbance of the single tyrosine residue at 274nm. The pH was maintained using 20mM phosphate buffer at pH 7. The samples were measured in a 1 or 2mm path length quartz cell (Starna).

To follow the effect of cosolute concentration on the protein folding equilibrium, the protein samples were titrated using concentrated cosolute stock solutions. The CD spectra of the titrated protein solutions were measured in quintuplicates from 190 or 200 to 260nm with 0.1nm steps at 25°C. The spectra were baseline-corrected by background subtraction of spectra of a solution with the same cosolute concentration but in the absence of protein. In addition, the ellipticities at 222nm for AQ16 and 215nm for MET16 were recorded and accumulated over 90 seconds. These accumulated ellipticities were used to determine the concentrations of the proteins' native, c_N, and denatured, c_D, states from the limiting

ellipticity values of AQ16's and MET16's native and denatured states.

For MET16, the native ellipticity of $-10,342$deg cm^2 dmol^{-1} was measured in 50%wt MeOH-water mixture,[S8] while a signal of 0 was used for the fully denatured protein.[S9–S11] For AQ16, the denatured ellipticity is 640deg cm^2 dmol^{-1}, while the native ellipticity depends on the protein length and is given by, $\theta_N = \theta_N^0\,(1 - 2.5/n)$, where θ_N is the protein's native state ellipticity, $\theta_N^0 = -42,500$deg cm^2 dmol^{-1} is the native state ellipticity of an infinitely long protein, and $n = 16$ is the number of AQ16's residues.[S12–S14] The folding free energy was then determined from the native and denatured states concentrations by,

$$\Delta G^0 = -RT \ln\left(\frac{c_N}{c_D}\right) \tag{S11}$$

where R is the gas constant.

Additionally, temperature scans were conducted at several cosolute concentrations. For each cosolute concentration the reversibility of the folding process was ensured by taking a full spectra measurement (190 or 200 to 260nm) in the beginning and end of each scan at 25°C. Temperature scan experiments ranged from 5 or 10°C to 60°C in 5°C steps and the folding free energy versus temperature was determined from accumulated ellipticities as described for the titration experiments.

The value of ε is determined by fitting the change in folding free energy, $\Delta\Delta G^0$ versus cosolute concentration, Fig. 3A, B of the main text. The fitting is preformed by solving the equilibrium condition, Eq. S7, with ε as the only variable, and using the previously determined values of ν and χ (sections S1.3 and S1.4). The resulting values of ε for AQ16 and MET16 with all cosolutes are in Fig. 5 and Table S4.

We dissect ΔG^0 into its enthalpic and entropic contributions by fitting the experimental ΔG^0 versus temperature to the integrated Van 't Hoff equation, Eq. 3, at different cosolute concentrations, Fig. 3C, D of the main text. The resulting change in folding enthalpy and entropy due to added sugars, $\Delta\Delta H^0$ and $\Delta\Delta S^0$, were further fitted to our mean-field model to resolve the enthalpic and entropic contribution to the soft-interaction parameter, ε_H and ε_{TS}, respectively. Here, the value of χ_{TS} as derived from experiments is added as an additional parameter, and the fit is performed with ε_{TS} as variable.

S1.6. Determination of Uncertainties in ε

The uncertainties of ε and ε_{TS} in Figs. 5, 8, and S8 and Table. S4 were determined by subsampling following the Monte Carlo cross-validation (MCCV) methodology. MCCV is usually used in validating the accuracy of fitted models, but it also allows estimation of variability of model parameters.[S15,S16] In accordance with MCCV, values from the experimental set of $\Delta\Delta G^0$ for a protein-cosolute pair were randomly sampled and assigned to a smaller

training set that comprises two-thirds the full $\Delta\Delta G^0$ set. By fitting our model to this training set we derived an estimate for the value of ε. This process was repeated $N = 100$ times, generating N estimates from which the uncertainties were determined. A similar procedure was applied for ε_{TS} with $N = 6$ considering the smaller data set of $\Delta\Delta H^0$ and $T\Delta\Delta S^0$.

S1.7. Concentration Dependence of ΔC_P

To determine the enthalpic and entropic contributions to the folding free energies we have fitted the experimental ΔG^0 versus temperature using Eq. 3, the integrated van 't Hoff equation. In the fits shown in Fig. 3C, D we have assumed that the change in heat capacity upon folding, ΔC_P, is independent of the identity and concentration of the cosolute, an assumption verified as follows.

Fig. S3A shows ΔC_P derived from fits to the data for AQ16 with each cosolute versus cosolute concentration, treating ΔC_P in Eq. 3 as a fitting parameter. Values of ΔC_P for the different cosolutes and all concentrations fluctuate with rather small and evenly distributed deviations around the mean value, $\langle \Delta C_P \rangle$. The positive value for the folding heat capacity of AQ16, $\langle \Delta C_P \rangle = 0.476 \pm 0.006 kJ/molK$, and the corresponding negative value for MET16, $-0.408 \pm 0.007 kJ/molK$, reflect the different thermodynamic folding mechanisms, as detailed in the main text.

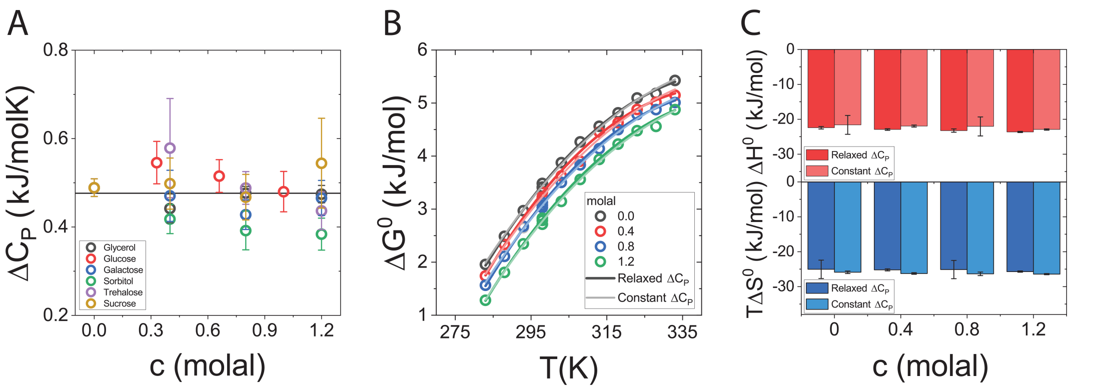

Figure S3: Concentration Dependence of ΔC_P for AQ16. **(A)** Values of ΔC_P versus cosolute concentration. The mean change in heat capacity, $\langle \Delta C_P \rangle$, is shown by the horizontal line. **(B)** AQ16's folding free energy versus temperature for different trehalose molal concentrations. Curves are fits to the integrated van 't Hoff equation, Eq. 3, with ΔC_P as a free (relaxed) fitting parameter or with constant $\Delta C_P = \langle \Delta C_P \rangle$. **(C)** AQ16's folding enthalpies and entropies for different trehalose concentrations from the fits in panel B.

Fig. S3B shows the van 't Hoff fits to ΔG^0 of AQ16 in presence of trehalose (same experimental data as in Fig. 3C) derived in two ways: (i) fits that treat ΔC_P as a fit parameter (as in panel A), shown as dark curves. (ii) fits to ΔG^0 where ΔC_P is fixed to

its mean values, $\langle \Delta C_P \rangle$ (full black line in Fig S3A), shown in the lighter colored curves. The two ways of fitting resulted in ΔH^0 and $T\Delta S^0$ that are almost identical, Fig. S3C. We conclude that treating the value of ΔC_P as a constant for all cosolutes at all concentrations does not change our results within experimental error.

S1.8. Graphics and Images

The proteins native state secondary structure, Fig. 1A were determined using PEP-FOLD3[S17] and rendered using PyMOL.[S18] Carbohydrate structural schemes, Fig. 1B, were rendered using ChemDraw Ultra 12.0.[S19]

S2. Values of Model Paramerters

Table S1 contains the values of $\Delta SASA$ of the proteins, Tables S2 to S4 contain the determined mean-field model parameters, and Table S5 contains the experimental $\Delta\Gamma_S$ for AQ16 and MET16 with each cosolute.

Table S1: Change in solvent accessible surface area, $\Delta SASA$, upon protein folding.

	$\Delta SASA$ (Å^2)
AQ16	242.6
MET16	419.0

Table S2: Excluded volume parameter, ν.^{a,b}

Parameter	Glycerol	Glucose	Galactose	Sorbitol	Trehalose	Sucrose
ν	3.95 (0.01)	6.27 (0.01)	6.26 (0.01)	6.71 (0.01)	11.7 (0.01)	11.9 (0.01)

^{a}Figures in brackets indicate the fit error.
^{b}Ratio of cosolute to water (0.018 L/mol) partial molar volumes.

Table S3: Non-ideal mixing parameters, χ.[a]

Paramter	Glycerol		Glucose		Galactose	
	kT/site	kJ L^{-1}	kT/site	kJ L^{-1}	kT/site	kJ L^{-1}
χ	0.233 (0.003)	32.1 (0.4)	0.317 (0.009)	44 (1)	0.35 (0.01)	48 (1)
χ_H	-0.25 (0.03)	-34 (4)	0.000 (0.009)	0.000 (1)	-0.72 (0.09)	-100 (10)
χ_{TS}	-0.48 (0.03)	-66 (4)	-0.317 (0.009)	-44 (1)	-1.07 (0.09)	-150 (10)
**Paramter**	**Sorbitol**		**Trehalose**		**Sucrose**	
	kT/site	kJ L^{-1}	kT/site	kJ L^{-1}	kT/site	kJ L^{-1}
χ	0.381 (0.001)	52.5 (0.1)	0.433 (0.004)	59.8 (0.5)	0.452 (0.001)	62.3 (0.1)
χ_H	0.09 (0.02)	12.4 (0.3)	-0.69 (0.09)	-100 (10)	-0.40 (0.09)	-60 (10)
χ_{TS}	-0.29 (0.02)	-40.0 (0.3)	-1.12 (0.09)	-150 (10)	-0.854 (0.09)	-120 (10)

[a]Figures in brackets indicate the fit error.

Table S4: Soft interaction parameter, ε.[a]

Protein	Paramter	Glycerol		Glucose	
		kT a^{-2}	kJ mol^{-1} nm^{-2}	kT a^{-2}	kJ mol^{-1} nm^{-2}
AQ16	ε	-0.032 (0.002)	-0.20 (0.01)	-0.038 (0.001)	-0.233 (0.006)
	ε_H	0.29 (0.03)	1.8 (0.1)	0.21 (0.01)	1.29 (0.06)
	ε_{TS}	0.32 (0.03)	2.0 (0.1)	0.25 (0.01)	1.54 (0.06)
MET16	ε	-0.049 (0.002)	-0.30 (0.01)	0.017 (0.003)	0.10 (0.02)
	ε_H	0.135 (0.007)	0.83 (0.04)	0.38 (0.04)	2.3 (0.2)
	ε_{TS}	0.184 (0.006)	1.13 (0.04)	0.36 (0.04)	2.2 (0.2)
**Protein**	**Paramter**	**Galactose**		**Sorbitol**	
		kT a^{-2}	kJ mol^{-1} nm^{-2}	kT a^{-2}	kJ mol^{-1} nm^{-2}
AQ16	ε	-0.029 (0.002)	-0.18 (0.01)	-0.061 (0.002)	-0.37 (0.01)
	ε_H	0.21 (0.01)	1.29 (0.06)	0.10 (0.02)	0.61 (0.02)
	ε_{TS}	0.24 (0.01)	1.47 (0.06)	0.16 (0.02)	0.98 (0.02)
MET16	ε	0.076 (0.002)	0.47 (0.01)	0.025 (0.003)	0.15 (0.02)
	ε_H	0.49 (0.01)	3.00 (0.06)	0.5 (0.04)	3.1 (0.1)
	ε_{TS}	0.41 (0.01)	2.53 (0.06)	0.47 (0.04)	2.9 (0.1)
**Protein**	**Paramter**	**Trehalose**		**Sucrose**	
		kT a^{-2}	kJ mol^{-1} nm^{-2}	kT a^{-2}	kJ mol^{-1} nm^{-2}
AQ16	ε	-0.045 (0.002)	-0.27 (0.01)	-0.069 (0.001)	-0.424 (0.006)
	ε_H	0.107 (0.002)	0.66 (0.02)	0.146 (0.001)	0.896 (0.006)
	ε_{TS}	0.152 (0.002)	0.93 (0.02)	0.215 (0.001)	1.320 (0.006)
MET16	ε	0.016 (0.003)	0.10 (0.02)	0.003 (0.003)	0.02 (0.02)
	ε_H	0.357 (0.009)	2.19 (0.05)	0.26 (0.02)	1.6 (0.1)
	ε_{TS}	0.341 (0.008)	2.09 (0.04)	0.26 (0.02)	1.6 (0.1)

[a]Figures in brackets indicate the error, determined by Monte Carlo analysis, see section S1.6.

Table S5: Change in preferential hydration coefficient, $\Delta\Gamma_S$, determined from model fits to the experimental $\Delta\Delta G^0$ at $c = 1$molal.

Protein	Glycerol	Glucose	Galactose	Sorbitol	Trehalose	Sucrose
AQ16	-5	-8	-9	-6	-17	-14
MET16	-6	-23	-32	-27	-40	-37

S3. Measured Solution Densities

Densities of aqueous cosolute binary solutions as a function of the cosolute molality at 25°C are given in Table S6. These values correspond to the densities shown in Fig. S1A. The cosolutes' molality was determined gravimetrically, section S1.3.

Table S6: Densities of aqueous mixtures at 25°C.

Glycerol		Glucose		Galactose	
molal	density (gr/ml)	molal	density (gr/ml)	molal	density (gr/ml)
0	0.99703	0	0.99703	0	0.99703
0.600	1.00940	0.599	1.03514	0.303	1.01749
1.221	1.02126	1.202	1.06829	0.603	1.03647
1.801	1.03097	1.801	1.09777	0.899	1.05397
2.420	1.04044	2.399	1.12318	1.201	1.07066
2.953	1.04819	2.995	1.14660	1.508	1.08666
3.544	1.05680	3.575	1.16609	1.807	1.10106
4.219	1.06457	4.197	1.18509	2.100	1.11555
4.747	1.07134	4.785	1.20100	2.403	1.12769

Sorbitol		Trehalose		Sucrose	
molal	density (gr/ml)	molal	density (gr/ml)	molal	density (gr/ml)
0	0.99703	0	0.99703	0	0.99703
0.599	1.03218	0.301	1.03498	0.600	1.06605
1.201	1.06274	0.599	1.06807	1.196	1.1206
1.805	1.08933	0.899	1.09765	1.797	1.16469
2.397	1.11215	1.200	1.12416	2.393	1.2006
3.010	1.13317	1.502	1.14797	2.999	1.23092
3.585	1.14983	1.801	1.16921	3.606	1.25639
4.168	1.16631	2.097	1.18823	4.198	1.27806
4.791	1.18159	-	-	4.797	1.29872

S4. Water Activities and Osmotic Pressures

Fig. S4 shows the cosolutes' osmotic pressure versus volume fraction at different temperatures. The osmotic pressures were calculated from measured water activities using Eq. S9 and these were fitted to the FH model using Eq. S10. The measured activity values are given in Tables S7–S12. Furthermore, the osmotic pressures at 25°C for each cosolute, measured using the vapor pressure osmometer (section S1.3), are shown in the insets of Fig. S4 and are given in Table S13. The good agreement between the measurements of our activity meter and osmometer verify the determined values of χ, which are independent of ν and ε. The standard deviations (given in brackets in Tables S7–S13) are derived from at least three measurements. The values of χ that result from the fits in Fig. S4 are given in Table S3, section S2.

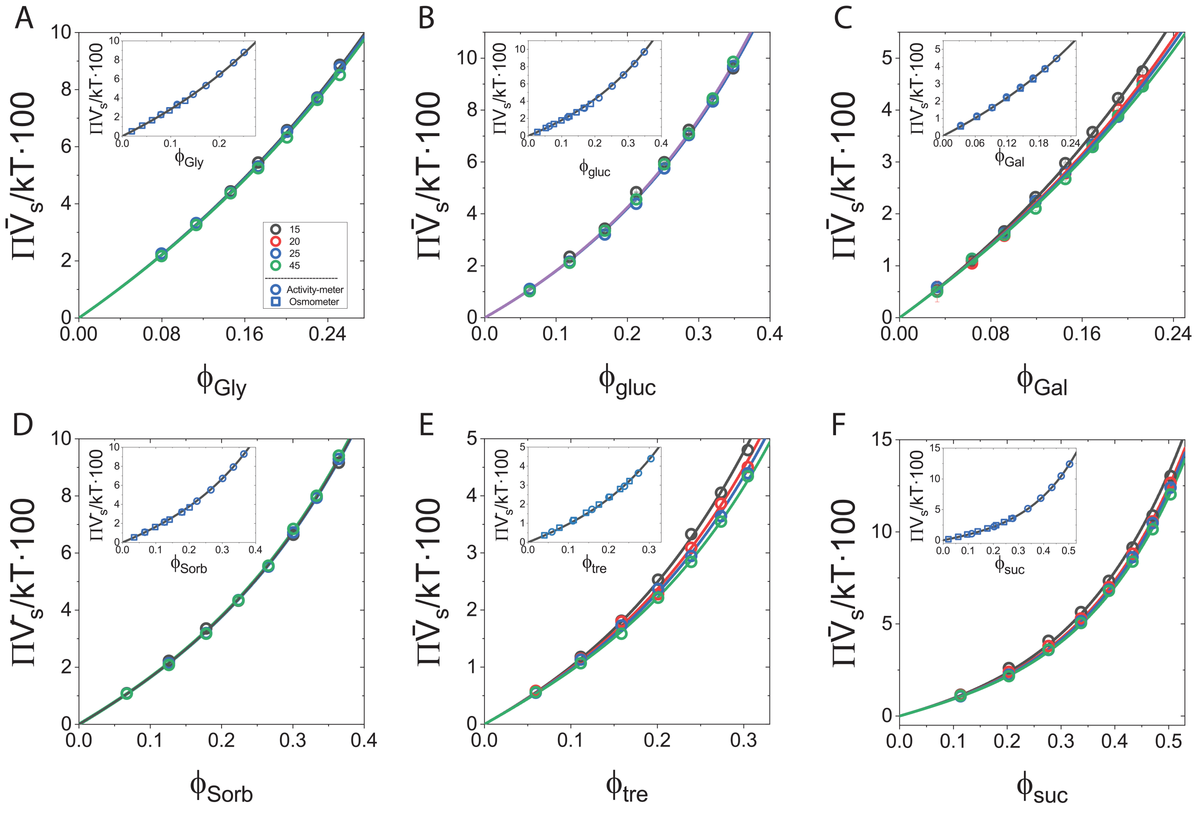

Figure S4: Scaled osmotic pressure versus volume fraction of **(A)** glycerol, **(B)** glucose, **(C)** galactose, **(D)** sorbitol, **(E)** trehalose, and **(F)** sucrose. Lines are fits to the FH osmotic pressure, Eq. S10. Insets compare the osmotic pressure that is derived from activity and osmometry measurements at 25°C.

Table S7: Water activity of aqueous glycerol mixtures at different temperatures.[a]

		a_S		
molal	ϕ_C	15°C	25°C	45°C
1.22127	0.07971	0.9778(0.0001)	0.9777(0.0003)	0.9785(0.0002)
1.80121	0.11325	0.9678(0.0002)	0.9673(0.0002)	0.9679(0.0006)
2.41994	0.14639	0.9566(0.0002)	0.9570(0.0002)	0.9572(0.0001)
2.95261	0.173	0.9470(0.0003)	0.9483(0.0004)	0.9488(0.0008)
3.54414	0.20077	0.9363(0.0003)	0.9368(0.0005)	0.939(0.001)
4.21888	0.22998	0.9256(0.0002)	0.9258(0.0001)	0.9264(0.0003)
4.74722	0.25161	0.9151(0.0003)	0.9158(0.0002)	0.9184(0.0005)

[a]Figures in brackets indicates the fit error.

Table S8: Water activity of aqueous glucose mixtures at different temperatures.[a]

		a_S		
molal	ϕ_C	15°C	25°C	45°C
0.59902	0.06317	0.990(0.001)	0.9890(0.0005)	0.9898(0.0004)
1.20166	0.11912	0.977(0.002)	0.9786(0.0002)	0.9790(0.0002)
1.80089	0.16849	0.9662(0.0005)	0.9684(0.0003)	0.9670(0.0004)
2.39899	0.21236	0.9527(0.0001)	0.9570(0.0004)	0.9554(0.0007)
2.99517	0.25178	0.9418(0.0002)	0.9441(0.0006)	0.9425(0.0003)
3.5745	0.28619	0.9302(0.0003)	0.9320(0.0002)	0.9315(0.0006)
4.19736	0.31971	0.9192(0.0001)	0.9200(0.0005)	0.9190(0.0004)
4.78534	0.34838	0.9084(0.0009)	0.9065(0.0005)	0.9050(0.0006)

[a]Figures in brackets indicates the fit error.

Table S9: Water activity of aqueous galactose mixtures at different temperatures.[a]

		a_S			
molal	ϕ_C	15°C	20°C	25°C	45°C
0.30271	0.03291	0.995(0.001)	0.995(0.002)	0.9941(0.0006)	0.9950(0.0005)
0.60315	0.06354	0.9891(0.0002)	0.9896(0.0005)	0.9887(0.0009)	0.9887(0.0002)
0.89884	0.09187	0.9835(0.0006)	0.9843(0.0006)	0.9839(0.0002)	0.9842(0.0004)
1.20129	0.11914	0.9770(0.0004)	0.9779(0.0003)	0.9778(0.0005)	0.9792(0.0003)
1.50826	0.14522	0.9706(0.0001)	0.9725(0.0002)	0.9723(0.0006)	0.9736(0.0003)
1.80669	0.16911	0.9649(0.0004)	0.9675(0.0004)	0.9672(0.0004)	0.9676(0.0004)
2.09971	0.1915	0.9586(0.0001)	0.9607(0.0002)	0.9620(0.0004)	0.9618(0.0007)
2.40341	0.21312	0.9536(0.0001)	0.95535(0.00005)	0.9562(0.0003)	0.9564(0.0007)

[a]Figures in brackets indicates the fit error.

Table S10: Water activity of aqueous sorbitol mixtures at different temperatures.[a]

		a_S		
molal	ϕ_C	15°C	25°C	45°C
0.59885	0.06721	0.989(0.003)	0.9892(0.0004)	0.9893(0.0008)
1.20087	0.12628	0.978(0.002)	0.9788(0.0004)	0.9794(0.0003)
1.80492	0.17845	0.967(0.001)	0.9685(0.0002)	0.9687(0.0005)
2.39694	0.22378	0.958(0.002)	0.9574(0.0001)	0.9575(0.0001)
3.01027	0.26569	0.946(0.002)	0.9462(0.0003)	0.9459(0.0005)
3.58469	0.30071	0.936(0.001)	0.9349(0.0005)	0.9338(0.0006)
4.16801	0.33324	0.9233(0.0008)	0.9237(0.0007)	0.9230(0.0003)
4.79054	0.36453	0.9124(0.0005)	0.9112(0.0001)	0.9101(0.0002)

[a]Figures in brackets indicates the fit error.

Table S11: Water activity of aqueous trehalose mixtures at different temperatures.[a]

		a_S			
molal	ϕ_C	15°C	20°C	25°C	45°C
0.30093	0.05933	0.994(0.002)	0.9942(0.002)	0.9945(0.0007)	0.99438(0.0002)
0.59881	0.11152	0.9883(0.0001)	0.989(0.001)	0.9887(0.0008)	0.98935(0.0007)
0.89877	0.15852	0.9820(0.0002)	0.9822(0.0005)	0.9828(0.0002)	0.98428(0.0003)
1.19995	0.20091	0.9750(0.001)	0.9775(0.0006)	0.9766(0.0003)	0.97808(0.0004)
1.50157	0.23923	0.9672(0.0009)	0.9696(0.0003)	0.9710(0.0003)	0.97195(0.0005)
1.80056	0.27367	0.9603(0.0002)	0.9621(0.0001)	0.9642(0.0007)	0.96513(0.0004)
2.09669	0.30475	0.9531(0.0006)	0.9559(0.0002)	0.957(0.001)	0.95747(0.0006)

[a]Figures in brackets indicates the fit error.

Table S12: Water activity of aqueous sucrose mixtures at different temperatures.[a]

		a_S			
molal	ϕ_C	15°C	20°C	25°C	45°C
0.6001	0.11332	0.9885(0.0004)	0.9887(0.001)	0.9893(0.0007)	0.98893(0.0005)
1.19623	0.20308	0.9743(0.001)	0.9762(0.0007)	0.9778(0.0002)	0.9785(0.0008)
1.79731	0.27673	0.9599(0.0005)	0.9626(0.0006)	0.9648(0.0002)	0.96455(0.0008)
2.39314	0.33724	0.9451(0.0001)	0.9483(0.0005)	0.9498(0.0004)	0.95073(0.0008)
2.99876	0.38894	0.9292(0.0003)	0.9323(0.0002)	0.9336(0.0003)	0.93423(0.0005)
3.60608	0.43297	0.9126(0.0005)	0.9156(0.0005)	0.9173(0.0003)	0.91957(0.0005)
4.19794	0.4701	0.8968(0.0005)	0.8996(0.0004)	0.9005(0.0005)	0.90352(0.0004)
4.79726	0.50351	0.8778(0.0004)	0.8810(0.0002)	0.8833(0.0002)	0.8866(0.0002)

[a]Figures in brackets indicates the fit error.

Table S13: Cosolute osmotic pressure from vapor pressure osmometer given in Osmolal.[a]

Glycerol		Glucose		Galactose	
molal	Π (Osm)	molal	Π (Osm)	molal	Π (Osm)
0.280	0.2783(0.0005)	0.246	0.243(0.001)	0.303	0.299(0.001)
0.600	0.612(0.003)	0.493	0.495(0.003)	0.603	0.607(0.002)
0.921	0.924(0.002)	0.740	0.751(0.002)	0.899	0.905(0.001)
1.221	1.246(0.003)	0.982	0.987(0.002)	1.201	1.213(0.003)
1.516	1.502(0.002)	1.251	1.250(0.001)	1.508	1.516(0.005)
1.801	1.802(0.002)	1.502	1.505(0.001)	1.807	1.811(0.003)
2.100	2.063(0.001)	1.780	1.789(0.002)	2.010	2.146(0.003)
-	-	2.053	2.046(0.002)	-	-

Sorbitol		Trehalose		Sucrose	
molal	Π (Osm)	molal	Π (Osm)	molal	Π (Osm)
0.300	0.294(0.001)	0.200	0.203(0.001)	0.100	0.0963(0.001)
0.599	0.593(0.001)	0.402	0.4258(0.0007)	0.300	0.305(0.002)
0.904	0.894(0.006)	0.607	0.650(0.002)	0.497	0.519(0.004)
1.201	1.180(0.006)	0.801	0.860(0.002)	0.750	0.798(0.004)
1.370	1.338(0.007)	1.015	1.102(0.001)	1.004	1.073(0.002)
1.805	1.762(0.001)	1.209	1.324(0.001)	1.251	1.356(0.002)
2.094	2.045(0.002)	1.411	1.555(0.002)	1.499	1.648(0.002)
		1.603	1.788(0.002)	1.755	1.966(0.003)

[a]Figures in brackets indicates the fit error.

S5. AQ16's CD Spectra in Presence of Trehalose

Fig. S5 shows the CD spectra of AQ16 for different concentrations of the dissacaride trehalose. The isodichroic point at 201 ± 1nm that is observed in temperature variation measurements, Fig. 2A, is conserved in the presence of trehalose, indicating that the structures of AQ16's native and denatured states do not change significantly upon addition of cosolutes.

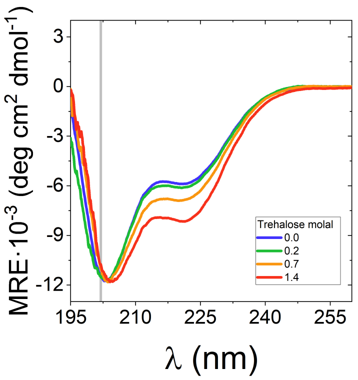

Figure S5: CD spectra of AQ16 for different trehalose concentrations at 25°C.

S6. Dissection of $\Delta\Delta G^0$ into Excluded Volume, Non-Ideal Mixing, and Soft-Interaction Contributions

Fig. S6 shows the contribution of $\Delta\Delta G^0_\nu$, $\Delta\Delta G^0_\chi$, and $\Delta\Delta G^0_\varepsilon$ to the folding free energy versus cosolute size, at a cosolute concentration of 1 molal. The stabilizing contribution of ν and destabilizing contribution of χ invariably scale with the cosolute size in both proteins. By contrast, $\Delta\Delta G^0_\varepsilon$ does not simply scale with cosolute size. For AQ16, $\Delta\Delta G^0_\varepsilon$ becomes more destabilizing with size for the monosaccharides and sorbitol but levels off for the larger disaccharides. For MET16 $\Delta\Delta G^0_\varepsilon$ changes from destabilizing for glycerol to stabilizing for the monosaccharides and sorbitol. For the larger disaccharides, trehalose and sucrose, the stabilizing contribution of ε decreases to almost zero.

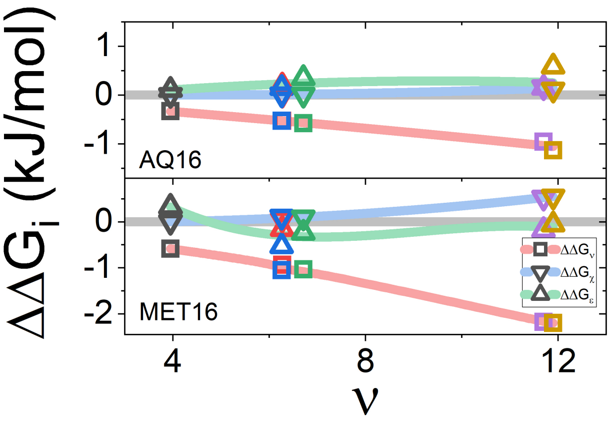

Figure S6: The free energy contributions, $\Delta\Delta G^0_\nu$, $\Delta\Delta G^0_\chi$, and $\Delta\Delta G^0_\varepsilon$ versus cosolute size, ν. The subscript i indicates the type of contribution associated with ν, χ, or ε. Data points represent values derived from model fits to data at cosolute concentration of 1 molal for **(A)** AQ16 and **(B)** MET16. The lines are used as a guide to the eye.

S7. Enthapic and Entropic Soft-Interaction Contributions are Strongly Compensating

Using our model, we can dissect the enthalpic and entropic contributions to $\Delta\Delta G^0_\nu$, $\Delta\Delta G^0_\chi$, and $\Delta\Delta G^0_\varepsilon$. Fig. S7 shows a representative example of these enthalpic and entropic contributions for the dissacaride trehalose. Although the trends in protein stability seen in Fig. S7 are also reflected in the $\Delta\Delta G^0$ of Fig. 7, the mapping of $\Delta\Delta G^0$ onto the enthalpy-entropy plane is useful, since it allows to visually distinguish the enthalpic and entropic terms and to asses the degree of their compensation.

The contributions of the excluded volume interactions are purely entropic, $\Delta\Delta G^0_\nu = -T\Delta\Delta S^0_\nu$, as they must always be, see vertical red lines. By contrast, the non-ideal mixing term is composed of a favorable enthalpic component, $\Delta\Delta H^0_\chi < 0$, and an unfavorable entropic component, $T\Delta\Delta S^0_\chi < 0$. The non-ideal mixing contributions, blue curves, are destabilizing since $T\Delta\Delta S^0_\chi < \Delta\Delta H^0_\chi$, and therefore reside below the diagonal that corresponds to $\Delta\Delta G^0 = 0$.

The purple curves in Fig. S7 show that the stabilizing effect of the joint contribution of ν and χ, $T\Delta\Delta S^0_\nu + T\Delta\Delta S^0_\chi$ versus $\Delta\Delta H^0_\chi$, is mostly entropic with a smaller stabilizing enthalpic contribution. Interestingly, the enthalpy-entropy signature of the sum contribution of ν and χ (but excluding the contribution of soft interactions) resembles the thermodynamic fingerprint that is often measured for polymeric crowders,[S20,S21] suggesting that soft interactions may play a lesser role in large polymeric crowders.

The enthalpy-entropy curves for the soft interaction contribution, $\Delta\Delta H^0_\varepsilon - T\Delta\Delta S^0_\varepsilon$, vary from residing above the diagonal for MET16 to below the diagonal for AQ16. This difference

between proteins seen in the entropy-enthalpy plot of the soft interaction contribution corresponds to the positive ε for MET16 and negative ε for AQ16. Nevertheless, we find that the soft interactions enthalpy-entropy curves are consistently close to the diagonal because the contributions of ε_H and ε_{TS} are strongly compensating.

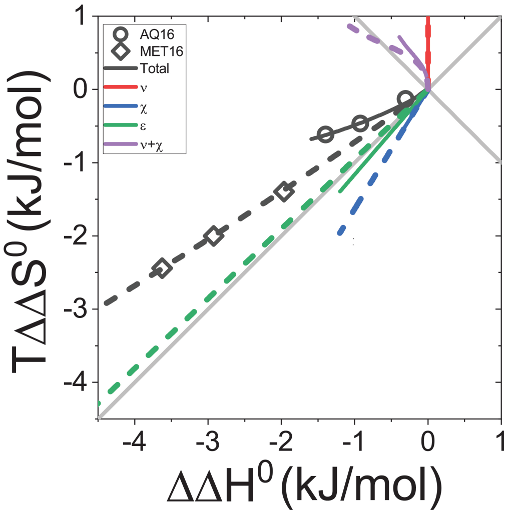

Figure S7: Enthalpic and entropic contributions to $\Delta\Delta G^0_\nu$, $\Delta\Delta G^0_\chi$, and $\Delta\Delta G^0_\varepsilon$ in presence of trehalose.

S8. Values of Protein-Sugar ε_H

Fig. S8 shows ε_H for AQ16 and MET16 with the sugar cosolutes. We find that $\varepsilon_H > 0$ for all cosolutes with both proteins, i.e., the enthalpic contribution to the soft interaction is repulsive.

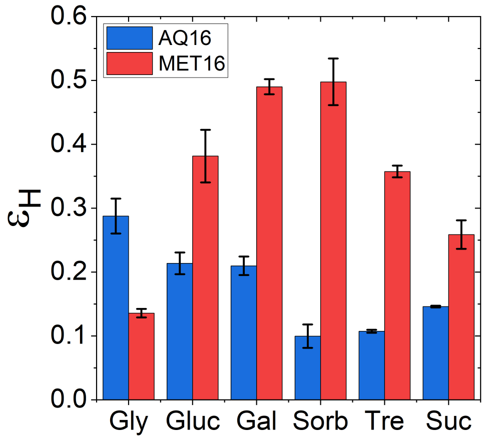

Figure S8: Values of ε_H for MET16 and AQ16 with sugar cosolutes.

S9. Simulation Details and Validation

The choice of a suitable simulation force field is crucial to properly describe the delicate interplay of forces between the solvent, cosolute, and protein in simulation, and to relate these molecular interactions to the experimental results.[S22,S23] Here we use the modified CHARMM36 force field developed by Cloutier et al[S24] because it reproduces the concentration dependence of $\Delta\Gamma_S$ for proteins in presence of carbohydrates,[S24] indicating that the forces between solvent, cosolute, and protein are well represented.

Molecular dynamic simulations were performed using the GROMACS package[S25] and the TIP3P water model.[S26] The Nosé-Hoover thermostat was utilized for temperature coupling,[S27,S28] particle-mesh Ewald was employed for electrostatic calculations,[S29,S30] and the LINCS method was used to fix the bond lengths to hydrogen atoms.[S31] Van der Waals interactions were truncated smoothly with a switching distance of 10Å and a cutoff distance of 13Å. All simulations ran for 200ns and converged after 100ns, as demonstrated by the protein radius of gyration, Fig. S9. All subsequent analyses were carried out on the second half of each trajectory.

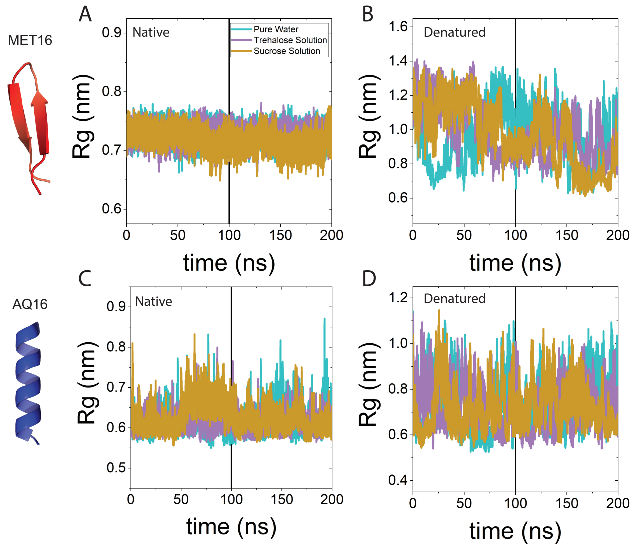

Figure S9: Radius of gyration versus simulation time of **(A)** MET16's native state, **(B)** MET16's denatured state, **(C)** AQ16's native state, and **(D)** AQ16's denatured state.

We validated our force field selection by comparing $\Delta\Gamma_S$ determined from simulations to $\Delta\Gamma_S$ determined from model fits to experiments, Table S14. Γ_S of the native and denatured states in simulation are determined from the relation,

$$\Gamma_S = N_S \left(1 - (n_S/n_C)/(N_S/N_C)\right) \tag{S12}$$

where N_S, N_C, n_S and n_C are the number of solvent (water) and cosolute (sugar) molecules

in the bulk and protein domain, respectively. Eq. S12 converges asymptotically to the value of Γ_S as the distance to boundary between protein and bulk domains, r, increases, Fig. S10A, B.[S32–S36] The difference in preferential hydration $\Delta\Gamma_S$ is calculated as the difference between the preferential hydration of the native and denatured states, $\Gamma_{S,N}$-$\Gamma_{S,D}$.

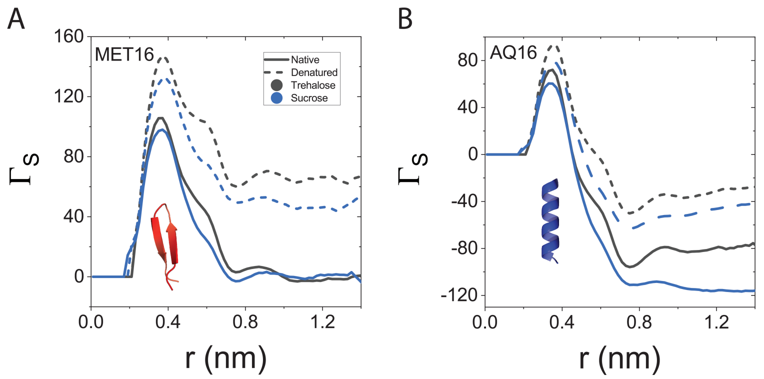

Figure S10: Change in preferential hydration parameter, $\Delta\Gamma_S$, due to protein folding from simulations of proteins in presence of trehalose or sucrose. **(A)** $\Delta\Gamma_S$ of MET16. **(B)** $\Delta\Gamma_S$ of AQ16.

Table S14: Changes in preferential hydration parameter, $\Delta\Gamma_S$, in presence of trehalose and sucrose from simulations and model fits to experiments. Figures in brackets indicate the standard deviation in Γ_S as determined in simulation.

	Simulations		Experiments[a]	
Protein	Trehalose	Sucrose	Trehalose	Sucrose
AQ16	-52 (3)	-74 (3)	-17	-14
MET16	-70 (2)	-45 (1)	-40	-37

[a]Values for $c = 1$molal

Although in simulation, MET16's values of $\Delta\Gamma_S$ are more negative than the experimentally determined values, Table S14, the values show the correct trend with respect to sugar identity and are in good qualitative agreement with the experimental $\Delta\Gamma_S$. Similarly, AQ16's values of $\Delta\Gamma_S$ from simulations are more negative than the experimental values, yet the trend with sugar identity is different from experiments, with trehalose showing less negative $\Delta\Gamma_S$ than sucrose in simulations but more negative $\Delta\Gamma_S$ in experiments. Thus, we find that for MET16 the simulation is qualitatively in good agreement with the experiments and for AQ16 the sign of $\Delta\Gamma_S$ determined from the simulations is in agreement with the experiments.

S10. Hydrogen Bonds: Methodology and Results

To probe the effect of added sugar on the hydrogen bond (Hbond) interactions of proteins with their environment, we employ our previously described methodology for evaluating the Hbond strength between pairs of donors and an acceptors in simulation.[S23,S37] This methodology has previously been used to analyze the Hbond network in aqueous mixtures of trehalose,[S23,S37] ethylene glycol,[S38] Nafion polymer,[S39] and liquid alcohol mixtures.[S40] Here, we extend the method beyond binary mixtures to include interactions of sugars and solvent molecules with protein backbone (bb) and side-chains (sd).

In our method, a free energy is associated with the formation of Hbonds, $\Delta\bar{G}_i$, between a donor-acceptor pair, i. $\Delta\bar{G}_i$ is determined from the probability distribution, $P_i(r,\theta)$, of donor-acceptor configurations with respect to donor-acceptor distance, r, and H-O/N$\cdots$H angle, θ. In the information-theoretic approach,[S41] $\Delta\bar{G}_i$, can be defined by the integral:

$$\Delta\bar{G}_i = -RT \int_{r_{min}}^{r_{max}} \int_0^{\pi} \xi_{rand} P_{i,rand}(r,\theta) \ln \frac{\xi_{rand} P_{i,rand}(r,\theta)}{\xi P_i(r,\theta)} d\theta dr \tag{S13}$$

where $P_{i,rand}(r,\theta)$ is the random distribution, calculated for each donor-acceptor pair numerically. ξ is the normalization constant of $P_i(r,\theta)$, and ξ_{rand} is the normalization constant of $P_{i,rand}(r,\theta)$ for the probability distribution between the integration boundaries of r_{min} and r_{max}, chosen to cover the relevant range for hydrogen bonding as given in Table S15.

The Hbonds strength and number of Hbonds are used to calculate the Hbond free energy using Eq. 4 of the main text. Fig. S11 shows the change in the proteins native and denatured states Hbond free energy due to addition of sugar. For MET16, the increased native state stability from backbone Hbonds, shown in Fig. 9, stems from interactions of water molecules

Table S15: Integration range for each Hbond pair

Hbond type	Integration range (Å)	Hbond type	Integration range (Å)
bb_N1W	2.6-3.2	sc_N1W	2.7-3.3
bb_N1H	2.7-3.3	sc_N1H	2.7-3.3
bb_N1O	2.7-3.5	sc_N1O	2.8-3.5
bb_COW	2.5-3.2	sc_COW	2.8-3.2
bb_COH	2.6-3.5	sc_COH	2.7-3.5
bb_HW	2.5-3.2	sc_HW	2.5-3.2
bb_HH	2.7-3.5	sc_HH	2.7-3.5
bb_HO	2.8-3.5	sc_HO	2.7-3.5
bb_N2W	2.6-3.5	sc_NpW	2.5-3.0
bb_N2H	2.7-3.5	sc_NpH	2.7-3.0
bb_N2O	2.7-3.5	sc_NpO	2.9-3.5

Table S16: Abbreviations used for Hbond pairs. Protein groups that participate in Hbonds are grouped as either backbone (bb) or side chain (sd). Hbond pairs that are found only in MET16, AQ16, or in both are shaded red, blue, and green, respectively.

	Abbriviation	Hbond pair
Backbone	bb_N2W	Secondary amide - Water
	bb_N2H	Secondary amide - Sugar hydroxyl
	bb_N2O	Secondary amide - Sugar ether
	bb_COW	Carbonyl - Water
	bb_COH	Carbonyl - Sugar hydroxyl
	bb_N1W	Primary amine - Water
	bb_N1H	Primary amine - Sugar hydroxyl
	bb_N1O	Primary amine - Sugar ether
Side chain	sc_HW	Hydroxyl - Water
	sc_HH	Hydroxyl - Sugar hydroxyl
	sc_HO	Hydroxyl - Sugar ether
	sc_N1W	Primary amine/amide - Water
	sc_N1H	Primary amine/amide - Sugar hydroxyl
	sc_N1O	Primary amine/amide - Sugar ether
	sc_COW	Carbonyl - Water
	sc_COH	Carbonyl - Sugar hydroxyl
	sc_NpW	Quaternary ammonium cation - Water
	sc_NpH	Quaternary ammonium cation - Sugar hydroxyl
	sc_NpO	Quaternary ammonium cation - Sugar ether

with the backbone's carbonyl and amide nitrogen groups, Fig. S11A. By contrast, the same water-backbone interactions destabilize AQ16's native state, Fig. S11C.

For interactions with the side-chains, the increased stabilization of MET16 mainly stems from water Hbonds with the charged amine of the lysine (sc_NpW) and the hydroxyl groups (sc_HW) of serine, threonine, and tyrosine, mainly because of their high abundance in MET16. The interactions of water with lysine's charged amine groups is shown to even become favorable in MET16's native state in presence of sugars, further adding to the native state stability, Fig. S11B. For AQ16, the modest contribution from the side-chains mainly originates in water Hbonds with glutamine's amide groups, while the Hbonds of water with the tyrosine's hydroxyl group are practically the same for the native and denatured states, Fig. S11D. However, this modest stabilizing contribution from interactions of water with glutamine's amide groups is insufficient to overturn the destabilizing Hbonds that include AQ16's backbone.

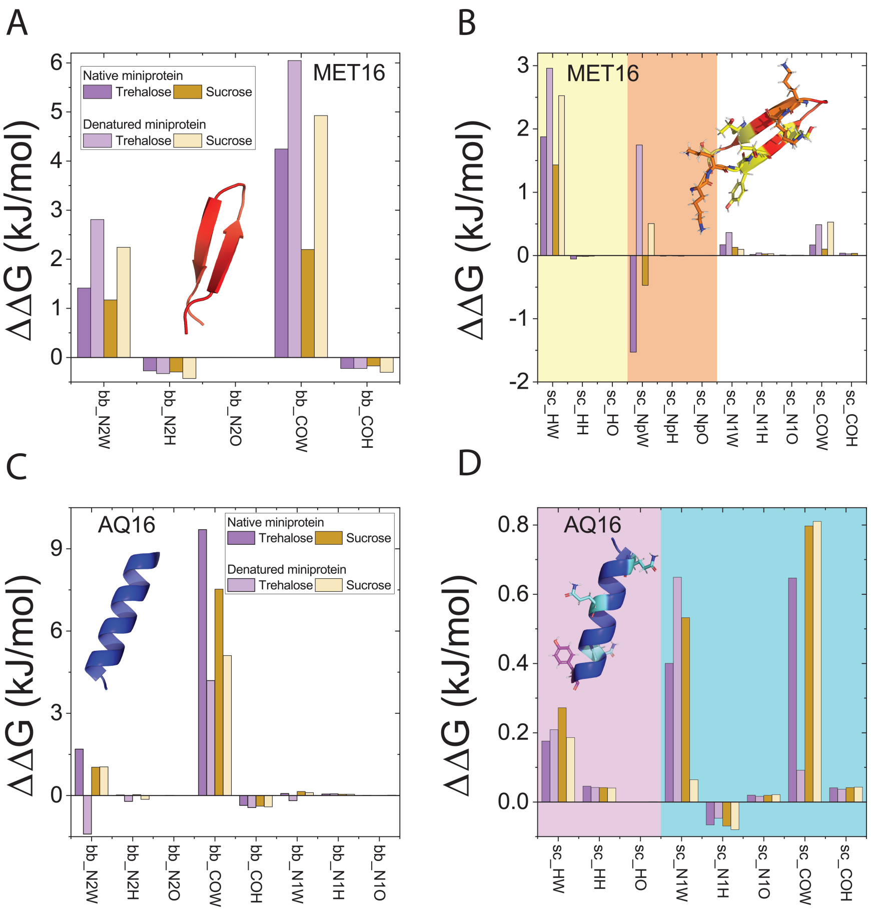

Figure S11: Dissected changes in Hbond free energy in presence of sugars. MET16's Hbond free energy for **(A)** backbone and **(B)** side-chains, and AQ16's Hbond free energy for **(C)** backbone and **(D)** side-chains. The schemes of MET16 and AQ16 in panels B and D highlight the side-chains that contribute most to the change in Hbond free energy. MET16's charged lysine amines are in orange and hydroxyls of serine, threonine, and tyrosine are in yellow. AQ16's amid group (carbonyl and nitrogen) of glutamine are in cyan and the hydroxyl of tyrosine are in purple. The background corresponds to the highlighted groups with the same colors.

References

(S1) Flory, P. J. Thermodynamics of high polymer solutions. *J. Chem. Phys* **1942**, *10*, 51–61.

(S2) Hill, T. L. *Introduction to Statistical Thermodynamics*; Dover Publications, 1986; pp 401–410.

(S3) Bernadó, P.; Blackledge, M.; Sancho, J. Sequence-specific solvent accessibilities of protein residues in unfolded protein ensembles. *Biophys. J* **2006**, *91*, 4536–4543.

(S4) Estrada, J.; Bernadó, P.; Blackledge, M.; Sancho, J. ProtSA: a web application for calculating sequence specific protein solvent accessibilities in the unfolded ensemble. *BMC Bioinf.* **2009**, *10*, 1–8.

(S5) Schellman, J. A. Protein stability in mixed solvents: a balance of contact interaction and excluded volume. *Biophys. J* **2003**, *85*, 108–125.

(S6) Sapir, L.; Harries, D. Is the depletion force entropic? Molecular crowding beyond steric interactions. *Curr. Opin. Colloid Interface Sci* **2015**, *20*, 3–10.

(S7) Sapir, L.; Harries, D. Macromolecular Stabilization by Excluded Cosolutes: Mean Field Theory of Crowded Solutions. *J. Chem. Theory Comput* **2015**, *11*, 3478–3490.

(S8) Olgenblum, G. I.; Wien, F.; Sapir, L.; Harries, D. β-Hairpin Miniprotein Stabilization in Trehalose Glass Is Facilitated by an Emergent Compact Non-Native State. *J. Phys. Chem. Lett* **2021**, *12*, 7659–7664.

(S9) Maynard, A. J.; Sharman, G. J.; Searle, M. S. Origin of β-hairpin stability in solution: structural and thermodynamic analysis of the folding of a model peptide supports hydrophobic stabilization in water. *J. Am. Chem. Soc* **1998**, *120*, 1996–2007.

(S10) Sukenik, S.; Politi, R.; Ziserman, L.; Danino, D.; Friedler, A.; Harries, D. Crowding Alone Cannot Account for Cosolute Effect on Amyloid Aggregation. *PLOS ONE* **2011**, *6*, e15608.

(S11) Politi, R.; Harries, D. Enthalpically driven peptide stabilization by protective osmolytes. *Chem. Commun* **2010**, *46*, 6449–6451.

(S12) Scholtz, J. M.; Qian, H.; York, E. J.; Stewart, J. M.; Baldwin, R. L. Parameters of helix–coil transition theory for alanine-based peptides of varying chain lengths in water. *Biopolymers* **1991**, *31*, 1463–1470.

(S13) Scholtz, J. M.; Barrick, D.; York, E. J.; Stewart, J. M.; Baldwin, R. L. Urea unfolding of peptide helices as a model for interpreting protein unfolding. *PNAS* **1995**, *92*, 185–189.

(S14) Rohl, C. A.; Chakrabartty, A.; Baldwin, R. L. Helix propagation and N-cap propensities of the amino acids measured in alanine-based peptides in 40 volume percent trifluoroethanol. *Protein Sci.* **1996**, *5*, 2623–2637.

(S15) Molinaro, A. M.; Simon, R.; Pfeiffer, R. M. Prediction error estimation: a comparison of resampling methods. *Bioinformatics* **2005**, *21*, 3301–3307.

(S16) Simon, R. *Fundamentals of data mining in genomics and proteomics*; Springer, 2007; pp 173–186.

(S17) Lamiable, A.; Thévenet, P.; Rey, J.; Vavrusa, M.; Derreumaux, P.; Tufféry, P. PEP-FOLD3: faster de novo structure prediction for linear peptides in solution and in complex. *Nucleic Acids Res.* **2016**, *44*, W449–W454.

(S18) Schrödinger, LLC

(S19) Cousins, K. R. Computer review of ChemDraw Ultra 12.0. *J. Am. Chem. Soc.* **2011**, *133*, 8388.

(S20) Sukenik, S.; Sapir, L.; Harries, D. Balance of enthalpy and entropy in depletion forces. *Curr. Opin. Colloid Interface Sci.* **2013**, *18*, 495–501.

(S21) Sukenik, S.; Sapir, L.; Gilman-Politi, R.; Harries, D. Diversity in the mechanisms of cosolute action on biomolecular processes. *Faraday Discuss.* **2013**, *160*, 225–237.

(S22) Ponder, J. W.; Case, D. A. Force fields for protein simulations. *Adv. Protein Chem.* **2003**, *66*, 27–85.

(S23) Olgenblum, G.; Sapir, L.; Harries, D. Properties of Aqueous Trehalose Mixtures: Glass Transition and Hydrogen Bonding. *J. Chem. Theory Comput.* **2020**, *16*, 1249–1262.

(S24) Cloutier, T.; Sudrik, C.; Sathish, H. A.; Trout, B. L. Kirkwood–Buff-derived alcohol parameters for aqueous carbohydrates and their application to preferential interaction coefficient calculations of proteins. *J. Phys. Chem. B* **2018**, *122*, 9350–9360.

(S25) Abraham, M. J.; Murtola, T.; Schulz, R.; Páll, S.; Smith, J. C.; Hess, B.; Lindah, E. Gromacs: High performance molecular simulations through multi-level parallelism from laptops to supercomputers. *SoftwareX* **2015**, *1-2*, 19–25.

(S26) Jorgensen, W. L.; Chandrasekhar, J.; Madura, J. D.; Impey, R. W.; Klein, M. L. Comparison of simple potential functions for simulating liquid water. *J. Chem. Phys.* **1983**, *79*, 926–935.

(S27) Nosé, S. A molecular dynamics method for simulations in the canonical ensemblet. *Mol. Phys.* **1984**, *52*, 255–268.

(S28) Hoover, W. G. Canonical dynamics: Equilibrium phase-space distributions. *Phys. Rev. A* **1985**, *31*, 1695–1697.

(S29) Darden, T.; York, D.; Pedersen, L. Particle mesh Ewald: An N·log(N) method for Ewald sums in large systems. *J. Chem. Phys.* **1993**, *98*, 10089–10092.

(S30) Essmann, U.; Perera, L.; Berkowitz, M. L.; Darden, T.; Lee, H.; Pedersen, L. G. A smooth particle mesh Ewald method. *J. Chem. Phys.* **1995**, *103*, 8577–8593.

(S31) Hess, B.; Bekker, H.; Berendsen, H. J.; Fraaije, J. G. LINCS: A Linear Constraint Solver for molecular simulations. *J. Comput. Chem.* **1997**, *18*, 1463–1472.

(S32) Athawale, M. V.; Dordick, J. S.; Garde, S. Osmolyte trimethylamine-N-oxide does not affect the strength of hydrophobic interactions: Origin of osmolyte compatibility. *Biophys. J.* **2005**, *89*, 858–866.

(S33) Shukla, D.; Shinde, C.; Trout, B. L. Molecular computations of preferential interaction coefficients of proteins. *J. Phys. Chem. B* **2009**, *113*, 12546–12554.

(S34) Gilman-Politi, R.; Harries, D. Unraveling the molecular mechanism of enthalpy driven peptide folding by polyol osmolytes. *J. Chem. Theory Comput.* **2011**, *7*, 3816–3828.

(S35) Shimizu, S.; Matubayasi, N. Preferential solvation: Dividing surface vs excess numbers. *J. Phys. Chem. B* **2014**, *118*, 3922–3930.

(S36) Sapir, L.; Harries, D. Wisdom of the crowd. *Bunsen-Magazin* **2017**, *19*, 152–162.

(S37) Sapir, L.; Harries, D. Revisiting hydrogen bond thermodynamics in molecular simulations. *J. Chem. Theory Comput.* **2017**, *13*, 2851–2857.

(S38) Jindal, A.; Vasudevan, S. Molecular Conformation and Hydrogen Bond Formation in Liquid Ethylene Glycol. *J. Phys. Chem. B* **2020**, *124*, 9136–9143.

(S39) Cui, R.; Li, S.; Yu, C.; Zhou, Y. The Evolution of Hydrogen Bond Network in Nafion via Molecular Dynamics Simulation. *Macromolecules* **2023**, *56*, 1688–1703.

(S40) Jindal, A.; Vasudevan, S. Hydrogen Bonding in the Liquid State of Linear Alcohols: Molecular Dynamics and Thermodynamics. *J. Phys. Chem. B* **2020**, *124*, 3548–3555.

(S41) Procaccia, I.; Levine, R. Potential work: A statistical-mechanical approach for systems in disequilibrium. *J. Chem. Phys.* **1976**, *65*, 3357–3364.
